# Direct Visualization of Peptide/MHC Complexes at the Surface and in the Intracellular Compartments of Cells Infected *In Vivo* by *Leishmania major*

**DOI:** 10.1371/journal.ppat.1001154

**Published:** 2010-10-14

**Authors:** Eric Muraille, Pierre Gounon, Julie Cazareth, Johan Hoebeke, Christoph Lippuner, Ana Davalos-Misslitz, Toni Aebischer, Sylviane Muller, Nicolas Glaichenhaus, Evelyne Mougneau

**Affiliations:** 1 Institut de Pharmacologie Moléculaire et Cellulaire, INSERM U924, Valbonne, France; 2 Université de Nice Sophia-Antipolis, Nice, France; 3 Institut de Pharmacologie Moléculaire et Cellulaire, UMR6097, Valbonne, France; 4 UPR 9021 CNRS, Institut de Biologie Moléculaire et Cellulaire, Strasbourg, France; 5 Vetsuisse Faculty, University of Zürich, Zürich, Switzerland; 6 Department of Nephrology, Hannover Medical School, Hannover, Germany; 7 Parasitology laboratory, Robert Koch Institute, Berlin, Germany; National Institutes of Health, United States of America

## Abstract

Protozoa and bacteria infect various types of phagocytic cells including macrophages, monocytes, dendritic cells and eosinophils. However, it is not clear which of these cells process and present microbial antigens *in vivo* and in which cellular compartments parasite peptides are loaded onto Major Histocompatibility Complex molecules. To address these issues, we have infected susceptible BALB/c (H-2^d^) mice with a recombinant *Leishmania major* parasite expressing a fluorescent tracer. To directly visualize the antigen presenting cells that present parasite-derived peptides to CD4^+^ T cells, we have generated a monoclonal antibody that reacts to an antigenic peptide derived from the parasite LACK antigen bound to I-A^d^ Major Histocompatibility Complex class II molecule. Immunogold electron microscopic analysis of *in vivo* infected cells showed that intracellular I-A^d^/LACK complexes were present in the membrane of amastigote-containing phagosomes in dendritic cells, eosinophils and macrophages/monocytes. In both dendritic cells and macrophages, these complexes were also present in smaller vesicles that did not contain amastigote. The presence of I-A^d^/LACK complexes at the surface of dendritic cells, but neither on the plasma membrane of macrophages nor eosinophils was independently confirmed by flow cytometry and by incubating sorted phagocytes with highly sensitive LACK-specific hybridomas. Altogether, our results suggest that peptides derived from Leishmania proteins are loaded onto Major Histocompatibility Complex class II molecules in the phagosomes of infected phagocytes. Although these complexes are transported to the cell surface in dendritic cells, therefore allowing the stimulation of parasite-specific CD4^+^ T cells, this does not occur in other phagocytic cells. To our knowledge, this is the first study in which Major Histocompatibility Complex class II molecules bound to peptides derived from a parasite protein have been visualized within and at the surface of cells that were infected *in vivo*.

## Author Summary

Phagocytosis is a cellular process that allows the engulfment of solid particles such as bacteria or parasites by the cell membrane and leads to the formation of an intracytoplasmic vesicle, the phagosome. Cell types that are capable of phagocytosis are called phagocytes and include monocytes, macrophages, dendritic cells and eosinophils. All these cells express Major Histocompatibility Complex class II molecules, although at different levels. These molecules allow the presentation of pathogen-derived peptides to CD4^+^ T lymphocytes, a mechanism that alerts the immune system of the occurrence of an infectious danger. In order to visualize the intracellular compartments in which complexes between pathogen-derived peptides and Major Histocompatibility Complex class II molecules are formed, we have generated a monoclonal antibody that reacts to a peptide derived from an immunodominant antigen of the intracellular parasite *Leishmania major* bound to a murine Major Histocompatibility Complex class II molecule. We have shown that these complexes are present on the phagosomes from various types of phagocytes but that only dendritic cells export these complexes to the plasma membrane, allowing the activation of pathogen-specific T cells.

## Introduction

The initiation of an adaptive immune response against a pathogen relies on the loading of microbial peptides onto Major Histocompatibility Complex (MHC) molecules in Antigen Presenting Cells (APCs) and on the recognition of these peptide/MHC complexes by T lymphocytes. Therefore, identifying the cell types that present peptide/MHC to T cells and the mechanisms that lead to the formation of these complexes is critical for understanding host-pathogen interactions. In contrast to soluble antigens that enter APCs through passive or receptor-mediated endocytosis, particles that are larger than 0.5 µm in size such as bacteria and protozoa enter host cells through phagocytosis [1]. This process has been evidenced, at least *in vitro*, in several cell types including macrophages, monocytes, dendritic cells (DCs), neutrophils and eosinophils, leading to the formation of pathogen-containing phagosomes in the cell [2]. In most cases, phagosomes fuse with endosomes and eventually with lysosomes leading to phagolysosomes. The final outcome of this process depends on the nature of the infected cell. In macrophages and neutrophils, phagolysosomes exhibit a low pH and contain high levels of proteases resulting in efficient pathogen degradation. DC phagolysosomes exhibit a higher pH and pathogen degradation is therefore reduced in these cells [3]. While the phagocytic activity of eosinophils has been recognized for a long time, almost nothing is known on their phagosomes. Of note, a number of pathogens have developed survival strategies to avoid degradation by phagocytic cells [4].

As phagosomes are part of the endocytic pathway, many studies have investigated the role of these organelles in the processing and presentation of pathogen-derived antigens. For this purpose, bone marrow-derived macrophages or DCs, as well as macrophage cell lines were incubated in *vitro* with either antigen-coupled latex beads or live or killed pathogens and further analyzed by confocal or electron microscopy. In some studies, latex beads phagosomes were purified due to their flotation capabilities and analyzed for their protein content by 2-D gels or Western Blotting. Results indicated that latex beads phagosomes expressed various molecules involved in antigen presentation such as MHC class I and II, H2-DM, the invariant chain (Ii), cathepsins and Transporters associated with Antigen Processing (TAP) [5]–[10]. Studies with bacteria- or protozoa- containing phagosomes have revealed a more complex situation. While MHC class II molecules have been evidenced on the phagosomes of murine peritoneal macrophages incubated with heat-killed *Listeria monocytogenes* [11], they were not found on *Legionella pneumophila-*containing phagosomes in human monocytes [12]. In the case of *Mycobacterium tuberculosis,* both MHC class I and class II molecules were detected on the phagosome membrane in human monocytes infected with both live and heat killed bacteria [13]. In agreement with these results, phagosomes purified from a human monocytic leukemia cell line that had been incubated with heat-killed *Mycobacterium tuberculosis* stimulated pathogen-specific T cell hybridomas to secrete IL-2 [14]. Other studies were performed using bone marrow-derived macrophages or DCs that were infected *in vitro* with the intracellular parasite *Leishmania major*. Data showed that *L. major* amastigotes settled in acidic parasitophorous vacuoles exhibiting all the features of phagolysosomes including the presence of lysosome markers such as LAMP-1, LAMP-2 and rab7p [15]. In addition, the phagosome membrane contained both MHC II and H-2M molecules suggesting that MHC/peptide complexes were assembled in the phagosome [15]. However, at least in macrophages, MHC class II molecules appeared to be retained in the phagolysosome and part of these molecules were internalized by amastigotes possibly preventing *L. major*-infected cells to present parasite as well as exogenous antigens [16], [17].

Here, we have sought to visualize peptide/MHC complexes at the surface and in intracellular compartments of various phagocytic cells infected with L. major in vivo. To this aim, we have generated a monoclonal antibody (mAb) that reacts with high avidity to a peptide derived from the *Leishmania* LACK antigen bound to I-A^d^. This antigen was chosen because it was immunodominant and expressed in amastigotes, and because it induced a strong protective immune response when used as a vaccine in susceptible BALB/c mice [18], [19]. Furthermore, a LACK immunodominant peptide has been identified, i.e. LACK_156-173_, and extensively characterized [20]. This mAb was successfully used to analyze the distribution of I-A^d^/LACK complexes at the surface and in the different intracellular compartments of various cell types that had been infected in vivo with *Leishmania major*.

## Results

### I-A^d^/LACK complexes can be visualized on the cell surface and within intracellular compartments using the mAb 2C44

To generate a mAb reacting to LACK_156–173_ bound to I-A^d^, we injected I-A^d^/LACK dimers into 16.2β T Cell Receptor (TCR) transgenic mice that exhibit an increased frequency of LACK-specific T cells as the result of the expression of the β chain of a LACK-specific TCR [21]. We expected CD4^+^ T cells from these animals to provide increased help to B cells possibly resulting in the generation of a higher number of plasmocytes and increasing the probability to obtain B cell hybridomas secreting mAbs reacting to I-A^d^/LACK. Lymph node (LN) cells from immunized mice were used to generate B cell hybridomas, and hybridoma supernatants were tested for the ability to stain I-A^d^-expressing fibroblasts pulsed with LACK_156–173_ but not OVA_323–339_. Four mAbs were obtained and further characterized for their specificity and avidity for their common I-A^d^/LACK ligand. Among these mAbs, 2C44 was the only one that stained LN cells from LACK-immunized BALB/c mice but not those from OVA-immunized mice (Supplementary Figure 1). Furthermore, 2C44 exhibited a very high avidity for I-A^d^/LACK (1.1 nM) with association (k_1_) and dissociation (k_−1_) rate constants of 6.64×10^4^ M^−1^ s^−1^ and 7.6×10^−5^ s^−1^ (Supplementary Table 1).

**Figure 1:**
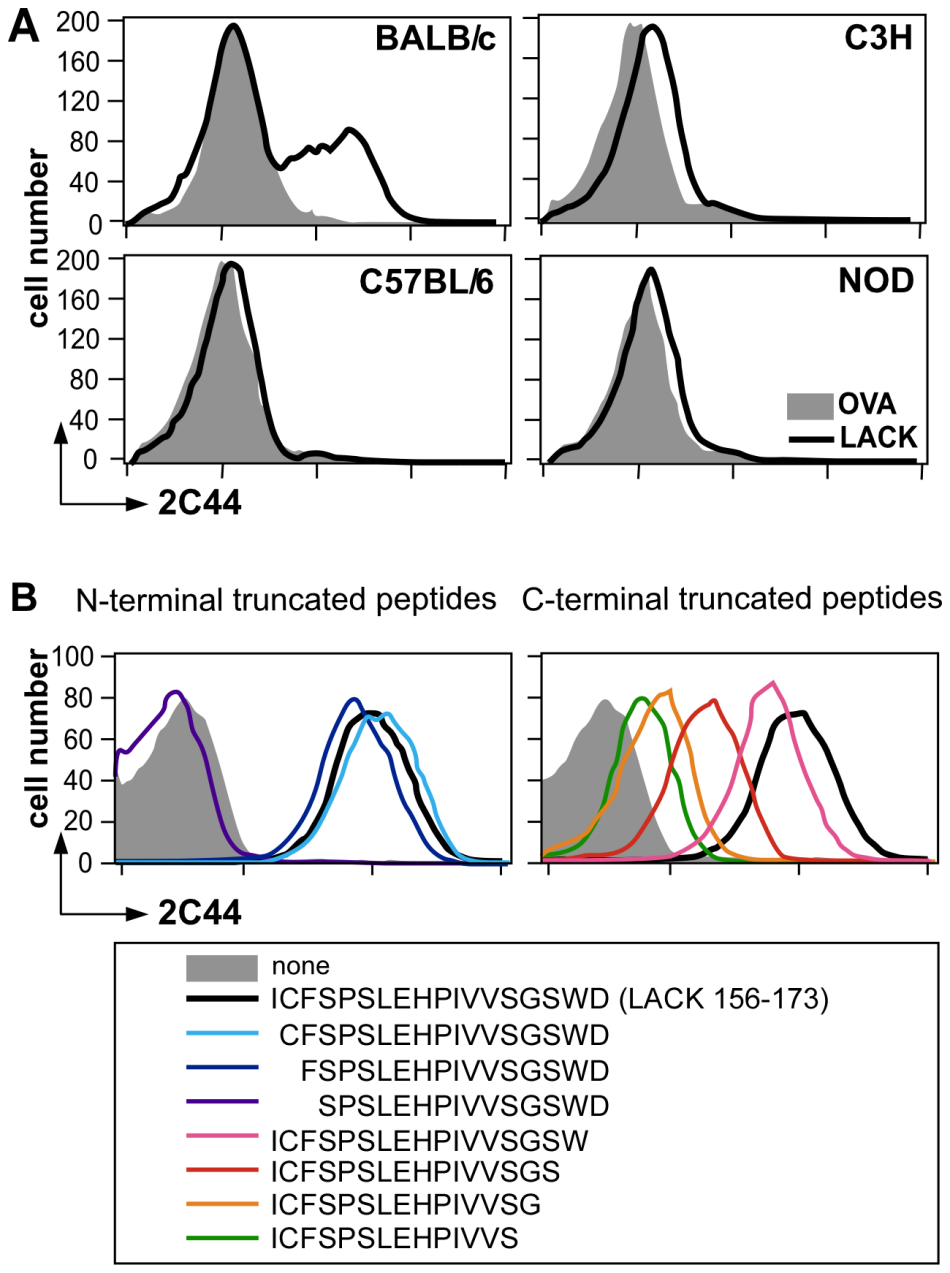
Visualization of I-A^d^/LACK complexes by flow cytometry. (A) Flow cytometry analysis of DCs purified from immunized mice. Mice of the indicated strains were injected into the hind footpads with 40 µg of LACK or OVA in CpG. LN cells were harvested 2 days later and enriched for CD11c^+^ cells using magnetic beads. Cells were stained with 2C44 and anti-CD11c mAb. Data show representative FACS profiles in mice immunized with LACK (solid line) or OVA (filled histogram). (B) Flow cytometry analysis of peptide-pulsed lymphoma cells. A20 mouse B lymphoma cells were pulsed or not with 1 µM of the indicated peptides and analyzed by flow cytometry upon staining with 2C44 and propidium iodide (PI). Data show representative FACS profiles after gating on PI^−^ cells.

**Figure 2:**
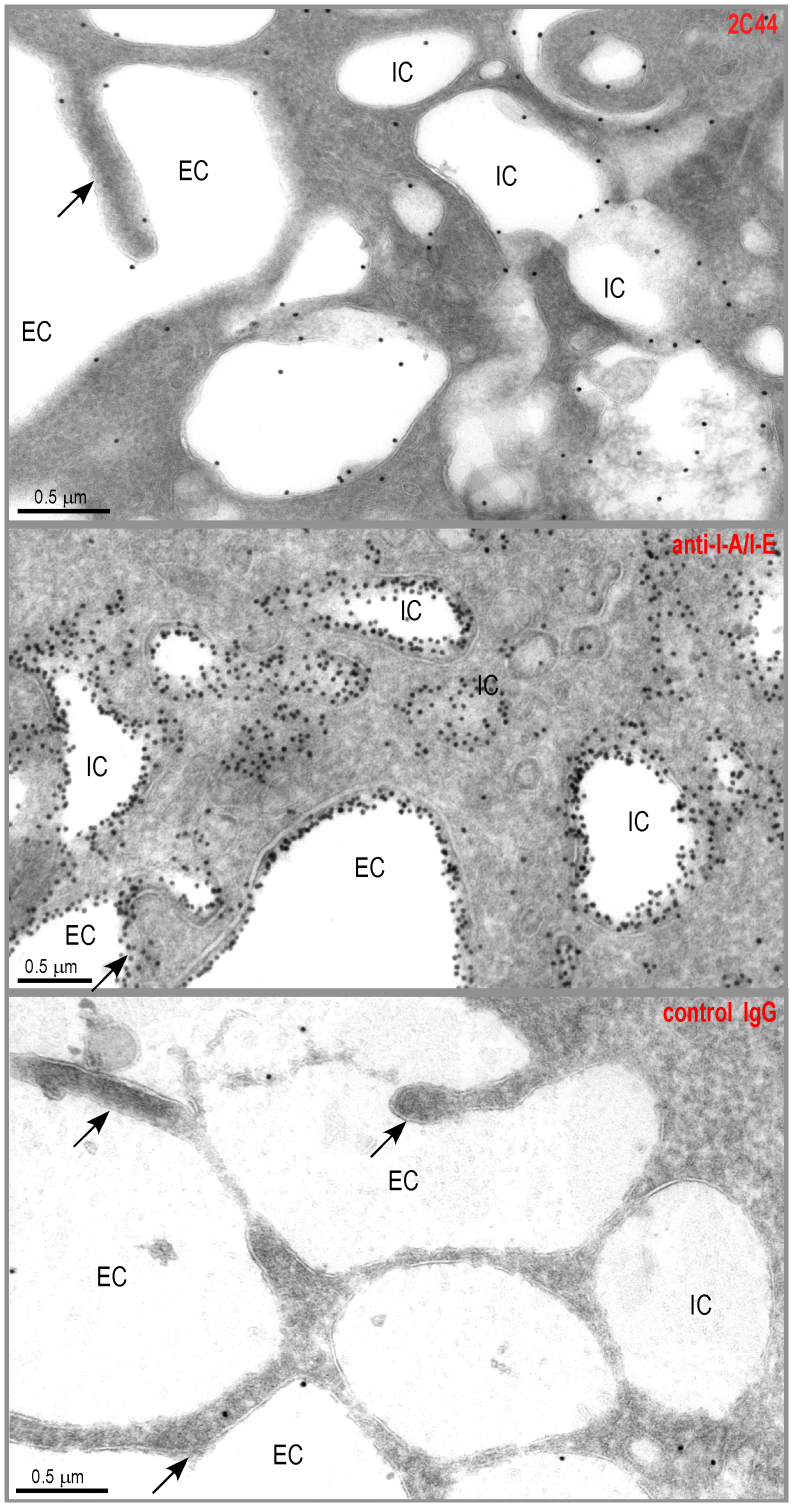
Visualization of I-A^d^/LACK complexes by immunogold electron microscopy. BALB/c mice were injected into the hind footpads with 40 µg of LACK in CpG. LN cells were harvested 2 days later and enriched for CD11c^+^ cells using magnetic beads. Cells were frozen and ultrathin cryosections were analyzed by electron microscopy after immunogold labelling with 2C44 (upper panel), anti-I-A/I-E (middle panel) or control isotypic (lower panel) mAbs. Representative photographs are shown. Intracellular Compartments (IC) and Extracellular Compartments (EC) are indicated. Arrows point to dendrites.

To further characterize the specificity of 2C44, BALB/c (H-2^d^), C3H (H-2^k^), C57BL/6 (H-2^b^) and NOD (H-2^g7^) mice were immunized with LACK or OVA. LN CD11c^+^ cells were purified 2 days later and analyzed by flow cytometry upon staining with 2C44. While 20–30% of CD11c^+^ cells were 2C44^+^ in LACK-immunized BALB/c mice, no staining or only background staining was observed in C3H, C57BL/6 or NOD mice immunized with LACK (Figure 1A). We next investigated whether 2C44 bound to I-A^d^-expressing A20 B lymphoma cells pulsed with truncated versions of LACK_156–173_. The binding of 2C44 was not affected by the removal of I_156_ or C_157_ but was completely prevented by the deletion of F_158_ (Figure 1B). Removal of one, two, three or four amino acids at the C terminal end of LACK_156–173_, resulted in a progressive decrease in the binding of 2C44, a result that could be explained by the reduced binding of these peptides to I-A^d^. We next investigated whether I-A^d^/LACK complexes could be visualized in intracellular compartments by immunogold electron microscopy. To this aim, BALB/c mice were immunized subcutaneously with either LACK or OVA in CpG. LN cells were purified 40 hours later, enriched for DCs using magnetic beads conjugated to anti-CD11c mAb, and fixed. Frozen sections were stained either with 2C44, an anti-I-A/I-E or a control isotypic mAb. Upon staining with 2C44, gold grains were evidenced both on the plasma membrane and on the inner membrane of intracellular compartments in LACK-immunized but not OVA-immunized mice (Figure 2 and data not shown). Only background staining was observed upon staining with an isotypic control mAb. As expected, staining with an anti-I-A/I-E mAb showed the presence of gold grains on the plasma membrane and in intracellular compartments at much higher levels than those observed with 2C44 (Figure 2).

### Different cell types contain amastigotes in the LN of *L. major*-infected mice

**Figure 3:**
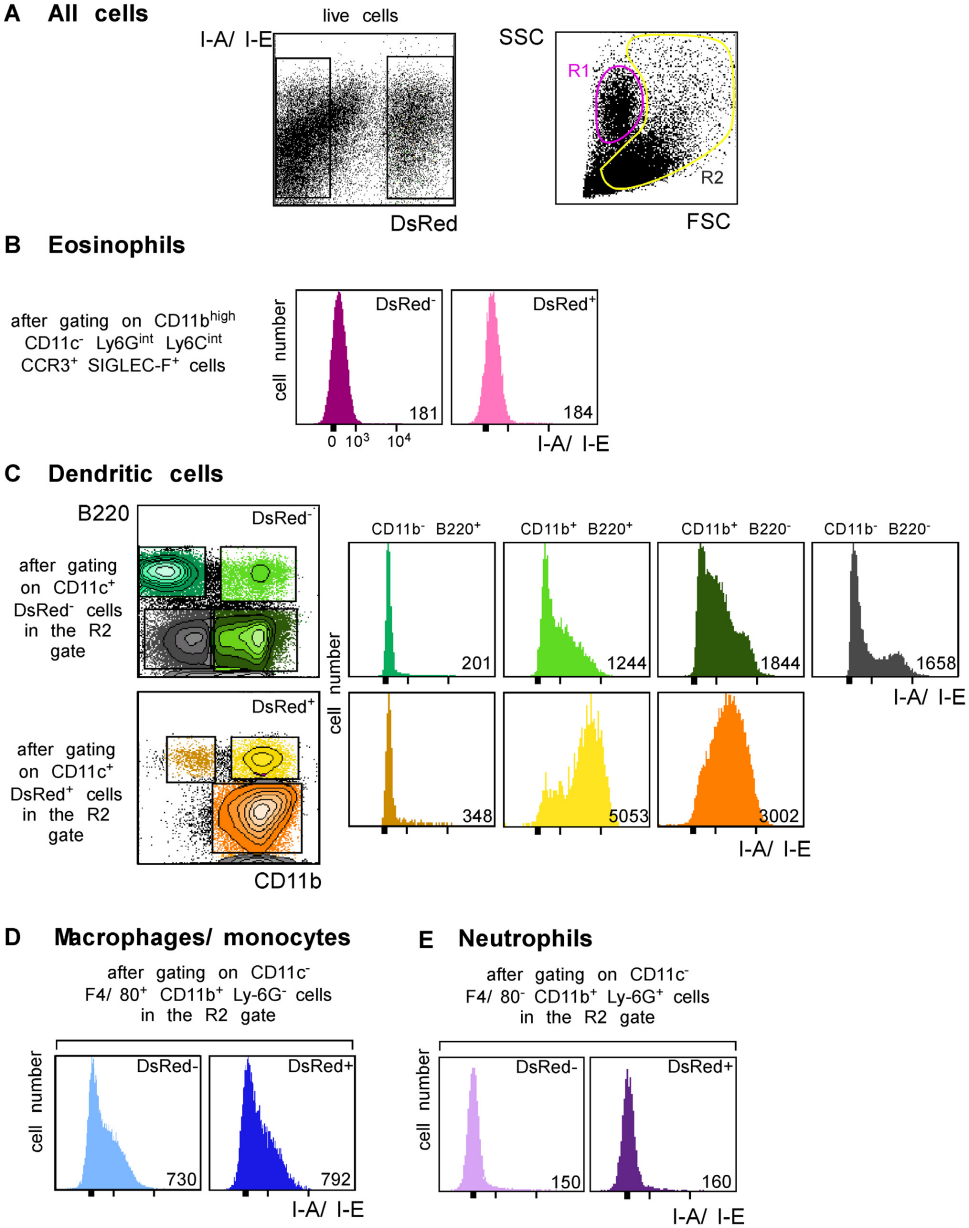
Flow cytometry analysis of LN cells. Lymphocyte-depleted LN cells from 4 wk-infected BALB/c mice were analyzed by flow cytometry after staining with mAbs directed to CD11c, CD11b, Ly-6C, Ly-6G, F4/80, Siglec-F, CCR3, B220 and I-A/I-E. Data show representative FACS profiles after gating on the indicated populations. Numbers indicate mfi. (A) All cells. The percentage of DsRed^+^ cells in the gated population is indicated (left panel). The right panel shows the R1 and R2 gates that were used later in this study. (B) Eosinophils. (C) Dendritic cells. (D) Macrophages/monocytes. (E) Neutrophils.

We next generated a fluorescent parasite that could allow for the purification of amastigote-containing cells. To this aim, we constructed a recombinant parasite in which the gene coding for a DsRed variant was inserted into the ribosomal RNA locus that is active in amastigotes [22]. BALB/c mice infected with DsRed-expressing parasites developed lesions as rapidly as those infected with wild type parasites demonstrating that parasite virulence was not impaired by DsRed expression (data not shown). To characterize infected cells, LN cells were purified from BALB/c mice that had been infected 4 wk earlier with DsRed-expressing promastigotes, depleted of both T and B lymphocytes, and analyzed by multicolor flow cytometry upon staining with antibodies directed to CD11c, CD11b, CD8α, Ly-6C, Ly-6G, F4/80, B220, Siglec-F and CCR3. DsRed^+^ profiles were readily observed in 8–12% of lymphocytes-depleted LN cells (Figure 3A, left panel). To visualize eosinophils, live cells were gated on CD11c^−^ CD11b^high^ Ly-6G^int^ Ly-6C^int^ Siglec-F^+^ CCR3^+^ cells. Further analysis showed that most (>95%) gated cells were FSC^low^ SSC^med/high^ and appeared in the R1 gate (Figure 3A). 15% of eosinophils were infected (Supplementary Figure 2) but all expressed low levels of MHC class II (Figure 3B). To visualize the other cell types, cells in the R2 gate (Figure 3A, right panel) were further gated depending on the expression of CD11c, CD11b, CD8α, B220, Ly6C, Ly6G and F4/80. DCs were identified as CD11c^+^ cells and 13% of them were infected (Supplementary Figure 3A, left panel). Four DC subsets were identified depending on the expression of CD11b and B220 (Figure 3C). CD11b^−^ B220^+^ DCs exhibited a typical plasmacytoid DC phenotype and 1.3% of these cells were DsRed^+^. Whether infected or not, these cells exhibited low surface levels of MHC class II. Among CD11b^+^ B220^+^ DCs, 31% were infected and expressed high levels of MHC class II while non-infected cells expressed low to intermediate levels of these molecules. CD11b^+^ B220^−^ DCs exhibited the phenotype of classical DCs and 25% were DsRed^+^. These cells expressed MHC class II with DsRed^+^ cells expressing higher levels of these molecules than DsRed^−^ cells. In contrast to the three other DC subtypes, CD11b^−^ B220^−^ DCs were all DsRed^−^. These cells were all CD8α^+^ and expressed heterogeneous levels of MHC class II. Macrophages/monocytes were identified as CD11c^−^ CD11b^+^ Ly-6G^low/int^ F4/80^high^ cells and 20% of these cells were infected (Supplementary Figure 3B). These cells expressed low to intermediate levels of MHC class II (Figure 3D). Neutrophils were identified as CD11c^−^ Ly-6C^int^ Ly-6G^high^ F4/80^neg^ cells and 25% of them were infected (Supplementary Figure 3C). Neutrophils did not express significant surface levels of MHC class II (Figure 3E). Further flow cytometry analysis showed that DCs, macrophages/monocytes, eosinophils and neutrophils accounted for 35%, 25%, 20% and 10% of DsRed^+^ cells respectively (data not shown).

**Figure 4:**
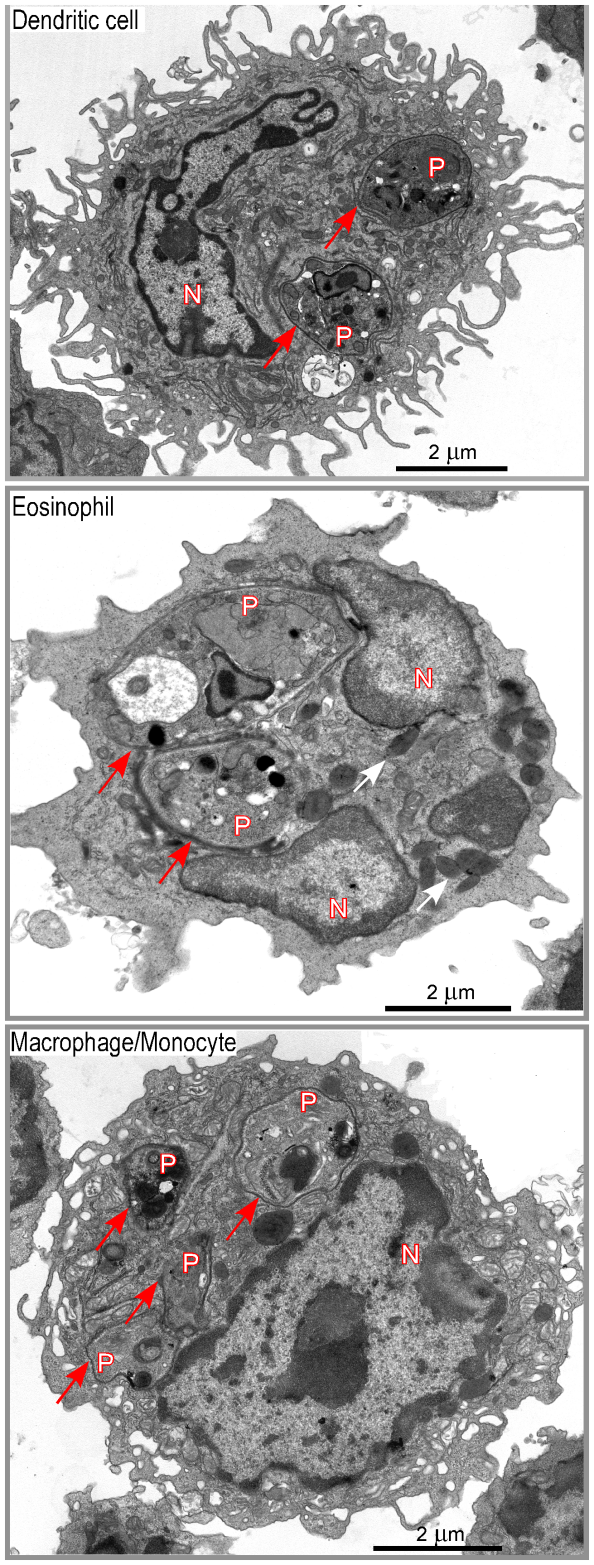
Morphological analysis of DsRed^+^ cells. Lymphocyte-depleted LN cells from 4 wk-infected BALB/c mice were analyzed by electron microscopy. Data show representative pictures of amastigote-containing DCs (upper panel), eosinophils (middle panel), and macrophage/monocytes (lower panel). Red and white arrows point to the phagosome membrane and to the eosinophil granules respectively. N: nucleus of the cell; P: phagosome.

DsRed^+^ cells were then sorted by flow cytometry, embedded with resin and analyzed by electron microscopy. In agreement with flow cytometry data, about 35% of sorted DsRed^+^ cells exhibited features of macrophages/monocytes such as irregular cytoplasmic projections or pseudopodia, cytoplasm with a few mitochondria, lysosomes and residual bodies (features that were not always all present on the same section), 20–25% of eosinophils (large ellipsoid granules) and 35–40% of DCs (thin and long dendrites) (Figure 4). Infected DCs contained an average of 1–3 amastigotes per section (data not shown). Each amastigote was localized within a parasitophorous vacuole and in most cases displayed an intact membrane, a nucleus containing diffuse and condensed chromatin with a nucleolus, golgi apparatus, mitochondria, mastigoneme and cortical microtubules (Supplementary Figure 4 and data not shown). These data strongly suggested that most amastigotes within DsRed^+^ DCs were alive. Infected eosinophils exhibited large ellipsoid granules and contained one to two amastigotes per section, each of them localized within a parasitophorous vacuole (Figure 4). As observed in DsRed^+^ DCs, amastigotes within infected eosinophils displayed recognizable and unaltered structures (data not shown). Infected macrophages exhibited a large number of vesicles and contained up to ten parasitophorous vacuoles per section, each one containing a single amastigote (Figure 4). In contrast to DCs and eosinophils, some macrophages contained partially degraded amastigotes with altered membranes and internal structures (data not shown).

### Infected DCs exhibit I-A^d^/LACK complexes both at their surface and on the phagosome membrane

**Figure 5:**
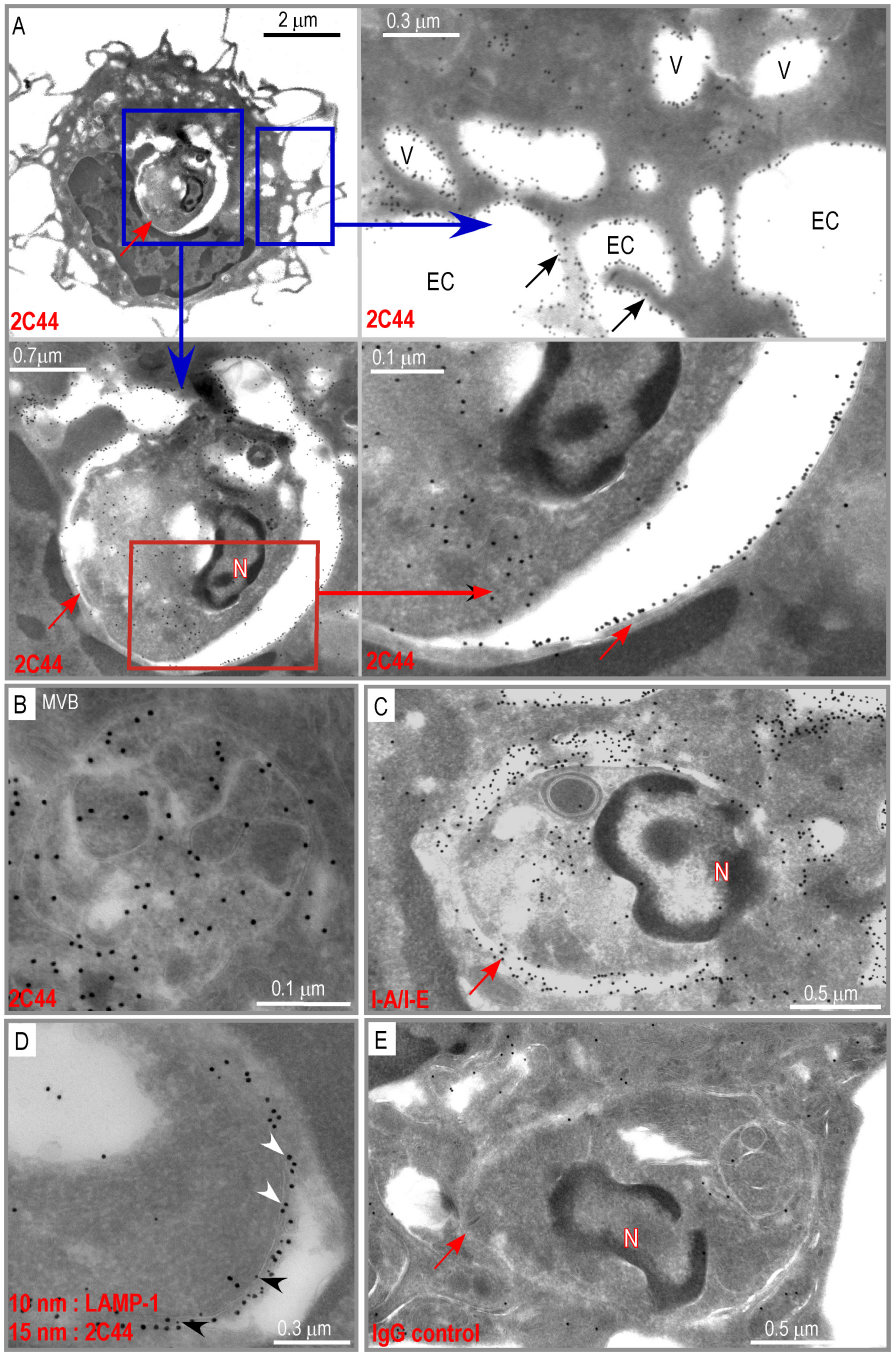
Distribution of I-A^d^/LACK complexes in intracellular DC compartments. Ultrathin cryosections of DCs purified from 4 wk-infected BALB/c mice were analyzed by immunogold electron microscopy after staining with either 2C44 (A, B), anti-I-A/I-E (C), a combination of a anti-LAMP-1 mAb (10 nm gold particles) and 2C44 (15 nm gold particles) (D), or a control isotypic mAb (E). (A) Data show different magnification views of a typical micrograph showing a DC harboring one single phagosome following staining with 2C44. Upper left panel: a low magnification view showing dendrites, the nucleus and an amastigote-containing phagosome. Lower left panel: a high magnification view showing the phagosome. Upper right panel: a high magnification view (after a 90° left rotation) showing gold grains located either on the cytoplasmic membrane or on small vesicles. Lower right panel: a high magnification view showing the two double sheets of the phagosome membrane with an intense 2C44 labelling of the inner side of the phagosomal membrane. (B) High magnification view showing of a small vesicle exhibiting gold grains both on peripheral and internal membranes. (C) An amastigote-containing phagosome stained with an anti-I-A/I-E mAb showing an intense labelling of the phagosomal membrane, small vesicles and the cytoplasmic membrane. (D) A fragment of an amastigote-containing phagosomal membrane labelled with both 2C44 (white arrowheads) and an anti-LAMP-1 mAb (black arrowheads). (E) An amastigote-containing phagosome stained with a control isotypic mAb. The red and black arrows point to the phagosome membrane and dendrites respectively. N: phagosome nucleus; EC: extracellular compartment; V: vesicles.

We next investigated whether I-A^d^/LACK complexes were present in phagolysosomes and/or in other cellular compartments. BALB/c and BALB.B mice were infected with DsRed-expressing promastigotes and sacrificed 4 weeks later. LN cells were depleted of both T and B lymphocytes and amastigote-containing cells were scored and analyzed for the presence of I-A^d^/LACK complexes using immunogold electron microscopy following staining with 2C44, anti-I-A/I-E, anti-LAMP-1 or control isotypic mAbs. Out of 115 scored amastigote-containing cells, 45 exhibited long dendrites but no granules indicating that they were DCs. Twenty-nine amastigote-containing cells exhibited eosinophil-specific features including large ellipsoid specific granules, with an electron-dense cristallin nucleus. Forty-one amastigote-containing cells did not show dendrites or granules but exhibited pseudopodia and a large number of vesicles suggesting that they were macrophage-related cells. In amastigote-containing DCs stained with 2C44, gold grains were readily detected on the plasma membrane, in phagosomes and on their membrane, and on much smaller cytoplasmic vesicles (Figure 5A). On the plasma membrane, gold grains were evenly distributed over the whole cell surface including dendrites. Within infected DCs, gold grains were found on the phagosome membrane and could clearly be evidenced on its inner side in some sections. Gold grains were also present within the amastigote. Gold grains were much less abundant and sometimes absent on the plasma and phagosome membranes of highly infected DCs (3–10 phagosomes per section) as compared to other DCs (1–2 phagosomes per section) (data not shown). This latter result suggested that the generation and/or the stability of I-A^d^/LACK complexes were influenced by parasite load and/or the DC physiological state. Most DCs also exhibited small cytoplasmic vesicles that did not contain parasite but exhibited gold grains mainly on their membrane. These vesicles exhibited a diameter of 0.2–0.5 µM and some of them exhibited features of multi vesicular bodies (MVB) including a round to oval shape with smaller vesicles inside and a double membrane (Figure 5B). Gold grains were also observed in all 2C44^+^ compartments upon staining with an anti-I-A/I-E mAb (Figure 5C). Co-staining with anti-LAMP-1 and 2C44 revealed that 2C44^+^ phagosomes as well as MVB-like vesicles expressed LAMP-1 (Figure 5D and data not shown). Only background staining was observed in BALB/c DCs stained with a control isotypic mAb and in BALB.B DCs stained with 2C44 (Figure 5E and data not shown). Altogether, our results unambiguously show that I-A^d^/LACK complexes are present at the surface of infected DCs as well as on the inner side of the phagosome membrane and of smaller vesicles that did not contain amastigotes.

### Infected macrophages and eosinophils exhibit I-A^d^/LACK complexes on the phagosome membrane but not at their surface

**Figure 6:**
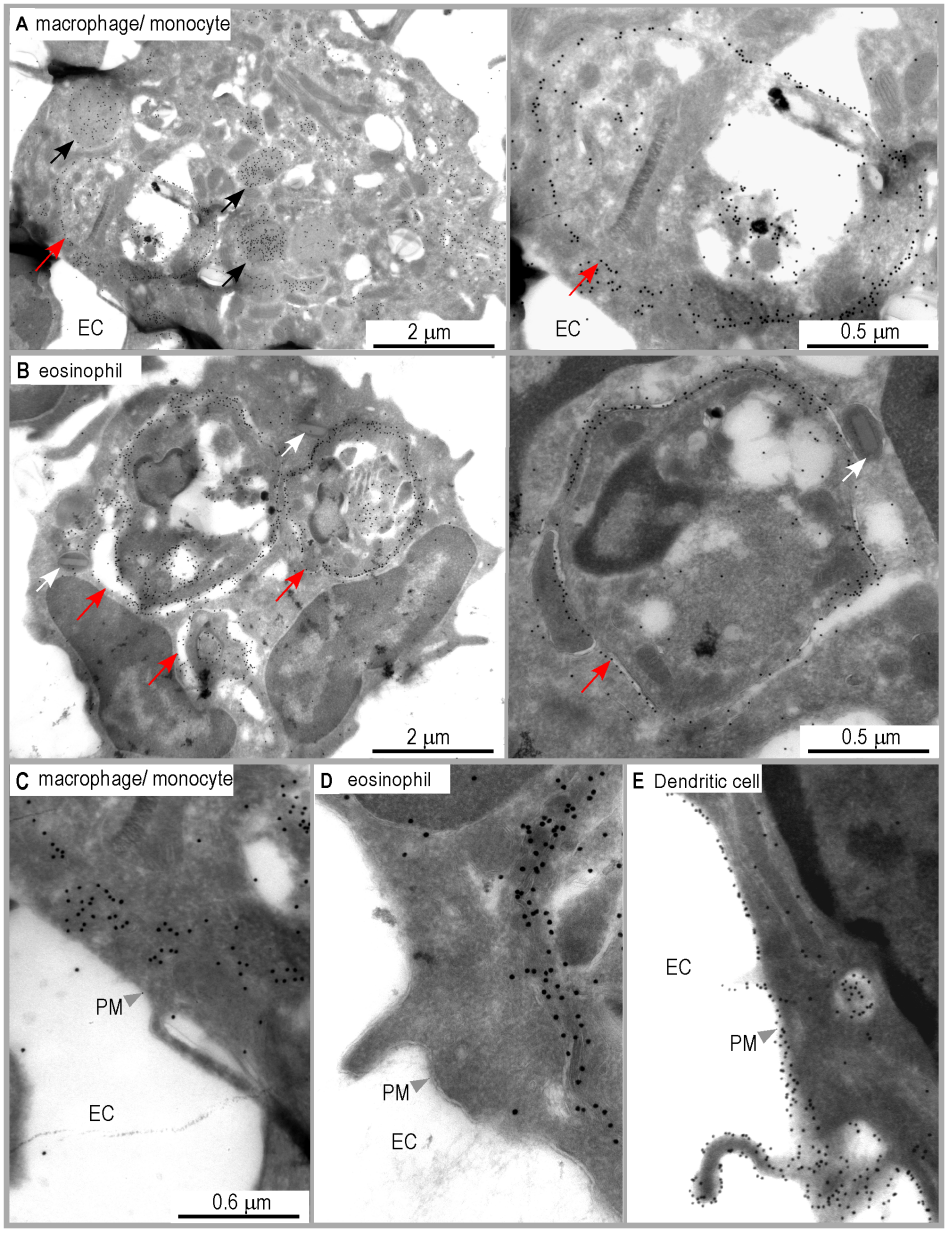
Distribution of I-A^d^/LACK complexes in eosinophil and macrophage intracellular compartments. Ultrathin cryosections of LN cells purified from 4 wk-infected BALB/c mice were analyzed by immunogold electron microscopy after staining with either 2C44. (A) A representative image of macrophages/monocytes. Left panel: a low magnification view of a macrophage exhibiting one amastigote-containing phagosome and several vesicles (black arrows). The photograph shows gold grains on the phagosome membrane and in the vesicles but not at the cell surface. Right panel: a high magnification view of the phagosome. Red arrows point to the phagosomal membrane. (B) A representative image of eosinophil. Left panel: a low magnification view showing an eosinophil containing three amastigote-containing phagosomes and typical ellipsoid granules (white arrows). The photograph shows gold grains on the phagosome membrane but not at the cell surface. Right panel: a high magnification view of an eosinophil phagosome. Red arrows point to the phagosomal membrane. (C, D, E) High magnification views of the plasma membrane in a representative macrophage/monocyte (C), eosinophil (D) and DC (E). EC: extracellular compartment. PM: plasma membrane.

Analysis of DsRed^+^ macrophages/monocytes by immunogold electron microscopy upon staining with 2C44 demonstrated the presence of gold grains in the phagosome and on the phagosome membrane, and in cytoplasmic vesicles, but not on the cell surface (Figure 6A and 6C). As compared to 2C44^+^ vesicles in DCs, 2C44^+^ vesicles in macrophages/monocytes were larger and exhibited diameters ranging from 0.2 to 1.0 µm. Some of the largest 2C44^+^ vesicles also exhibited electron-dense structures similar to *Leishmania* kinetoplast suggesting that they were phagosomes in which the amastigote was being killed (data not shown). Of note, and in contrast to those observed in phagosomes, gold grains were homogeneously distributed in large 2C44^+^ vesicles but only scarcely on their membrane.

Unexpectedly, gold grains were also observed in amastigote-containing eosinophils upon staining with 2C44 (Figure 6B and 6D), but not with a control isotypic mAb (data not shown). In these cells, gold grains were only observed in the phagosome and on the phagosome membrane. Co-staining revealed that 2C44^+^ phagosomes also displayed LAMP-1 (data not shown). Furthermore, staining with an anti-I-A/I-E mAb revealed that MHC class II molecules were present on the phagosome membrane but not on the plasma membrane (data not shown).

Altogether, these results suggest that macrophages/monocytes and eosinophils can capture *L.* major amastigotes, process parasite antigens and display MHC/peptide complexes on the phagosome membrane, but are unable to transport these complexes to the cell surface.

### Quantitative analysis of I-A^d^/LACK complexes on the plasma and the phagosome membranes

**Figure 7:**
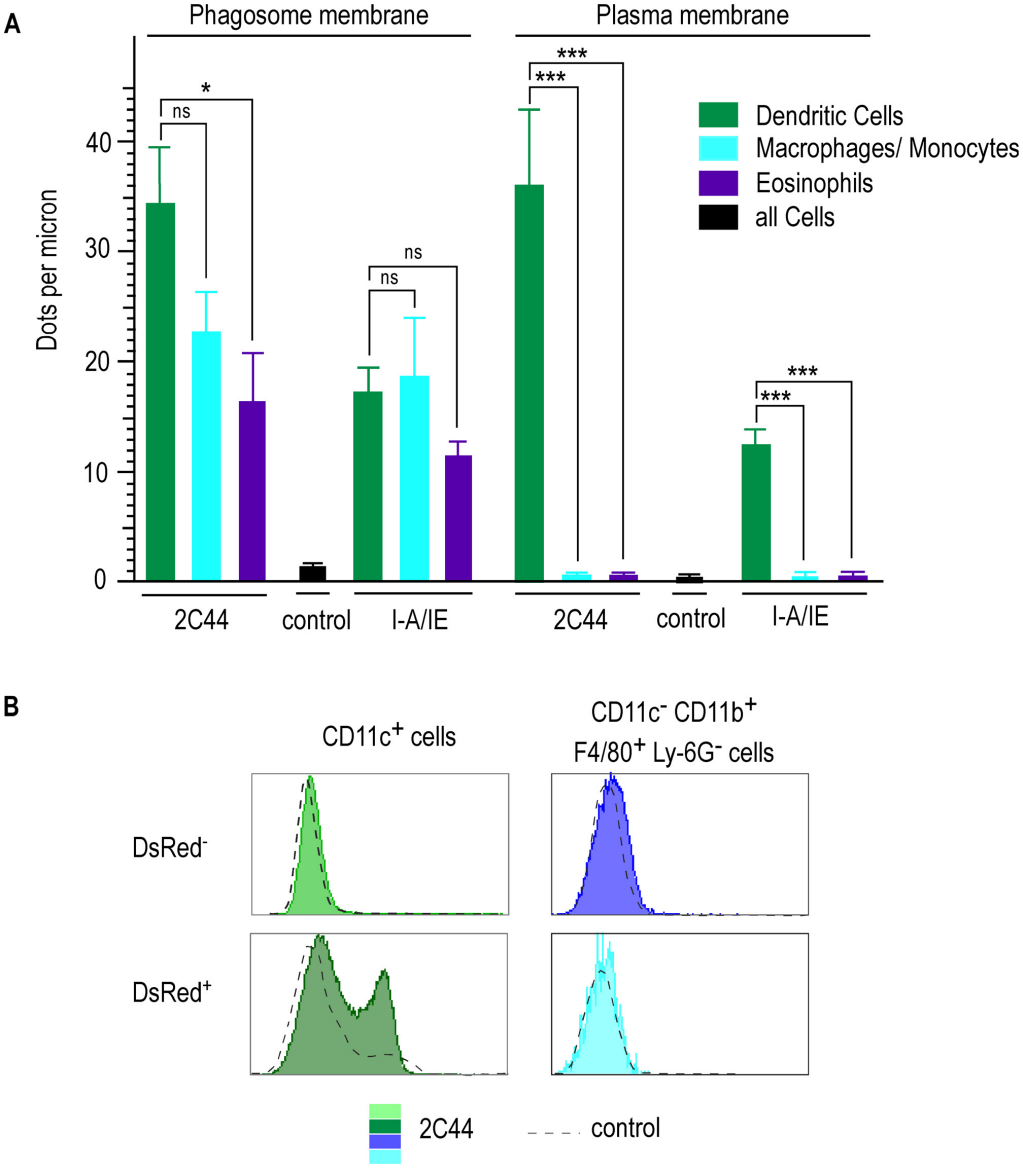
Quantitative analysis of I-A^d^/LACK complexes on the phagosome and the plasma membranes in infected phagocytes. Lymphocyte-depleted cells from 4 wk-infected BALB/c mice were enriched for DsRed^+^ cells. (A) Number of dots per micron. Cells were analyzed by immunogold electron microscopy after staining with 2C44, an anti-I-A/I-E or a control mAb. Cell types were identified according to their morphology and the number of dots per micron of membrane was counted. At least 30 sections were analyzed for each cell type and each staining. Data show the mean +/− s.e.m. (B) Flow cytometry analysis. Cells were analyzed by FACS after staining with 2C44 (filled histograms) or a control Ig (dotted line). Data show representative profiles after gating on CD11c^+^ cells (left panels) or CD11c^−^ CD11b^+^ F4/80^+^ Ly6G^−^ cells (right panels).

To obtain quantitative data, we counted the number of gold grains per micron on the phagosome and the plasma membranes in different cell types upon staining with 2C44, anti-I-A/I-E or a control mAb (Figure 7A). Upon staining with 2C44, amastigote-containing DCs exhibited 30-40 dots per micron on both the phagosome and the plasma membranes. The number of dots per micron on the plasma and the phagosome membranes was also similar upon staining with anti-I-A/I-E mAb and 20–50 fold higher than with a control mAb. In contrast to DCs, the number of dots per micron in macrophages/monocytes stained with 2C44 was much higher on the phagosome membrane than on the plasma membrane (22.5+/−3.9 versus 1.4+/−0.5, p<0.0001). Similar data were observed upon staining with an anti-I-A/I-E mAb. Likewise, the number of dots per micron in eosinophils stained with 2C44 was 16.4+/−1.4 on the phagosome membrane as compared to 0.35+/−0.06 on the plasma membrane (p<0.0001).

As another method to estimate the levels of I-A^d^/LACK complexes at the cell surface, lymphocyte-depleted LN cells from 4 wk-infected BALB/c mice were analyzed by flow cytometry after staining with antibodies directed to various surface markers and 2C44 or a control isotypic Ig (Figure 7B). While DsRed^−^ DCs exhibited the same profile upon staining with 2C44 and a control Ig, DsRed^+^ DCs exhibited a bimodal distribution upon staining with 2C44 with 30% of these cells that were 2C44^+^. In contrast to DCs, macrophages/monocytes, eosinophils and neutrophils, whether infected or not, exhibited the same profile upon staining with 2C44 and a control Ig (Figure 7B and data not shown).

**Figure 8:**
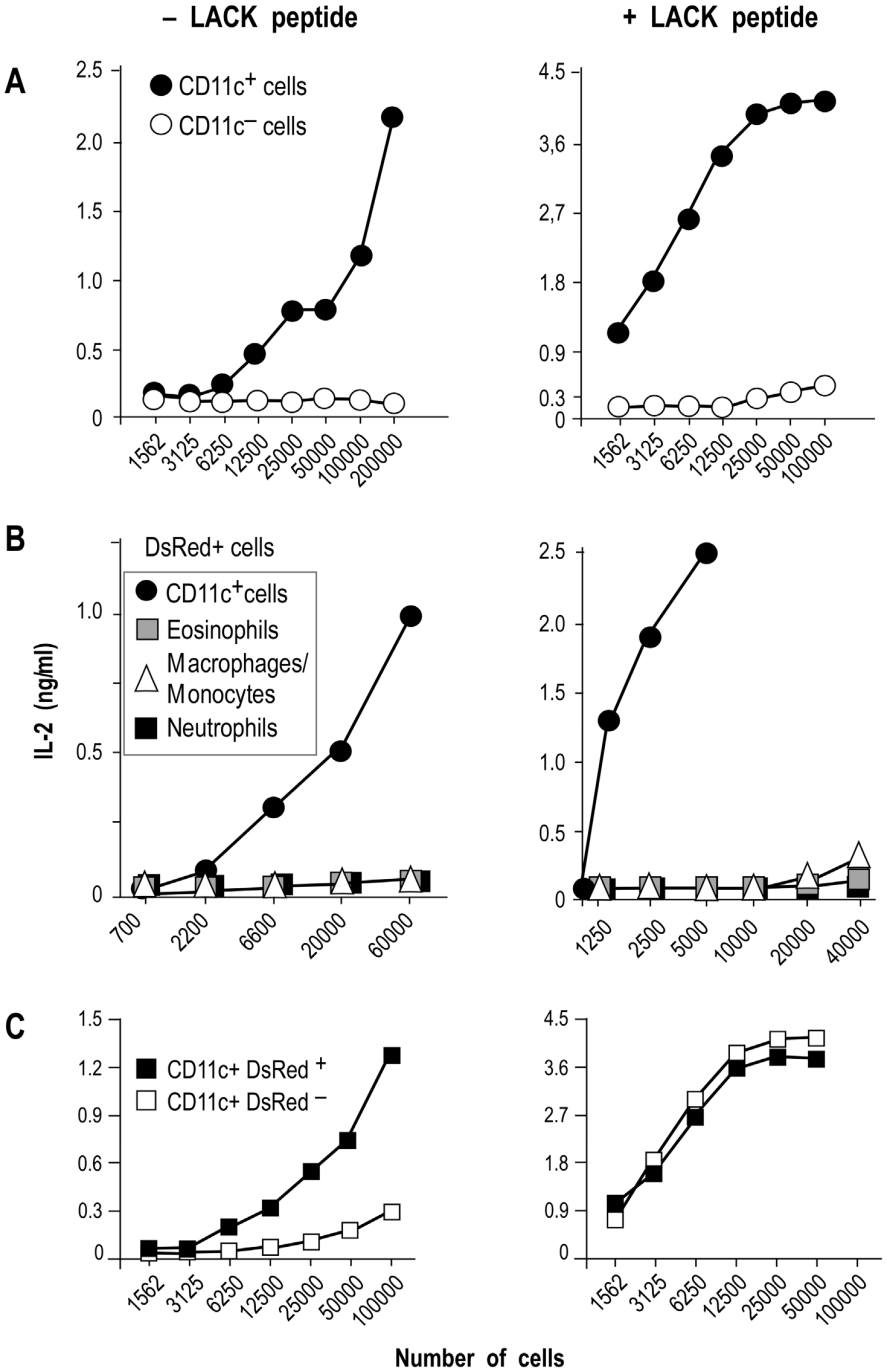
IL-2 secretion by LACK-specific T cell hybridomas upon incubation with different phagocytes. Lymphocyte-depleted cells from 4 wk-infected BALB/c mice were sorted into different cell types and the indicated numbers of cells were incubated with 10^5^ LMR7.5 T cell hybridomas with or without 1 µM of LACK_156–173_. Supernatants were harvested 24 hours later and analyzed for IL-2 content by ELISA. (A) Cells were sorted into CD11c^+^ (filled circles) and CD11c^−^ (empty circles) cells. Data show the results of a typical experiment (out of 5). (B) DsRed^+^ populations: CD11c^+^ (filled circles), eosinophils (grey squares), macrophages/monocytes (empty triangles) neutrophils (filled squares). Data show results of a typical experiment (out of 3). (C) CD11c^+^ cells were sorted into DsRed^+^ cells (filled squares) and DsRed^−^ cells (filled squares). Data show results of a typical experiment (out of 4).

To independently estimate the amount of I-A^d^/LACK complexes at the cell surface, lymphocytes-depleted LN cells from 4 wk-infected BALB/c mice were sorted into different cell types and incubated with the highly sensitive LACK-specific LMR7.5 hybridoma (Figure 8). In contrast to CD11c^+^ cells, CD11c^−^ cells did not stimulate LMR7.5 hybridomas to secrete IL-2 in the absence of added LACK peptide (Figure 8A). In the presence of peptide, CD11c^+^ cells induced high levels of IL-2 while CD11c^−^ cells induced only very low levels at the highest APCs/T cell ratio. We also tested the ability of different DsRed^+^ and DsRed^−^ cell populations to stimulate LMR7.5 hybridomas. In the presence or in the absence of LACK peptide, DsRed^+^ DCs induced LMR7.5 hybridomas to secrete relatively high amounts of IL-2, while DsRed^+^ eosinophils and DsRed^+^ neutrophils failed to do so. DsRed^+^ macrophage/monocytes induced very low levels of IL-2 at the highest APCs/T cell ratio and only in the presence of LACK peptide (Figure 8B). As expected, neither DsRed^−^ eosinophils nor DsRed^−^ macrophage/monocytes or neutrophils induced IL-2 secretion (data not shown). However, low levels of IL-2 were secreted by LMR7.5 hybridomas in the presence of DsRed^−^ DCs without added LACK peptide (Figure 8C) suggesting that these cells display low levels of I-A^d^/LACK complexes at their cell surface. Of note, these low levels could not be detected when DsRed^−^ DCs were analysed by flow cytometry after staining with 2C44, a result that was not unexpected, given the high sensitivity of LMR7.5 hybridomas.

Altogether, our data demonstrated that I-A^d^/LACK complexes were present at the surface of infected DCs as well as on the phagosome membrane. In contrast, infected eosinophils and infected macrophages/monocytes both exhibited I-A^d^/LACK complexes on the phagosome membrane, but not on the plasma membrane.

## Discussion

Here, we have tried to visualize MHC class II molecules loaded with a pathogen-derived peptide at the surface and in the intracellular compartments of phagocytic cells that had been infected with *Leishmania major in vivo*. To achieve this goal, we have generated a recombinant parasite expressing a fluorescent tracer and a mAb reacting to an antigenic peptide derived from the parasite LACK antigen bound to I-A^d^ MHC molecules. Amastigotes-containing DCs, macrophages/monocytes and eosinophils were readily identified by both flow cytometry and electron microscopy. Among these phagocytes, DCs accounted for 35% of all infected cells. In this study, we have classified DCs into four subsets depending on the expression of CD11b and B220. Among these subsets, CD11b^−^ B220^+^, CD11b^+^ BB20^+^, CD11b^+^ B220^−^, but not CD11b^−^ B220^−^, exhibited DsRed^+^ profiles. While the frequency of DsRed^+^ cells among CD11b^+^ B220^+^ and CD11b^+^ BB20^−^ were 31% and 25% respectively, only 1.3% of CD11b^−^ B220^+^ were DsRed^+^. Our data are in agreement with a previous study in which both B220^+^ DCs and B220^−^ DCs were found to be infected [23]. However, while Baldwin and colleagues concluded from their data that a significant proportion of plasmacytoid DCs were infected, we found that only 1.3% of these cells were DsRed^+^. This discrepancy may be explained by the fact that Baldwin and colleagues considered all B220^+^ CD11c^+^ cells to be plasmacytoid DCs while we could discriminate between *bona fide* CD11b^−^ B220^+^ DCs and a non described CD11b^+^ B220^+^ DCs subset that exhibit the specific feature of up-regulating MHC class II upon infection with *L. major*.

It remains to be determined why all DC subsets are not equally infected with *Leishmania*. It is conceivable that amastigotes enter all DCs but that the various DC subsets differ in their ability to support parasite replication. Alternatively, since the uptake of *L. major* by DCs is mediated by Fcγ receptors RI and RIII [24], it is possible that these receptors are differentially expressed by the different DC subsets. In agreement with this latter hypothesis, a molecule that is involved in the phagocytosis of *Leishmania major* parasites, SIRP-β, was found to be expressed on CD8α^−^ but not on CD8α^+^ DCs [25]. As CD11b^−^ B220^−^ DCs are all CD8α^+^, this may explain why we found that CD11b^−^ B220^−^ DCs were not infected.

While DCs accounted for 35% of infected cells in BALB/c mice in the present study, as many as 70–75% of infected cells were identified as DCs in C57BL/6 mice 4 weeks after infection [26], [27]. Furthermore, Leon *et al.* found that amastigote-containing eosinophils accounted for 2–3% of infected cells in C57BL/6 mice while 20–25% of infected cells were eosinophils in the present study [26]. One might speculate that differences in parasite load between susceptible and resistant mice could account for these distinct infected cells profiles.

One of the major findings of this paper is that various types of amastigote-containing phagocytes express I-A^d^/LACK complexes in the phagosome and on its inner membrane but that only DCs export these complexes at their surface. Immunogold electron microscopy data revealed that in DCs, I-A^d^/LACK complexes were present on the plasma membrane and evenly distributed over the whole cell surface including dendrites. I-A^d^/LACK complexes were also present in amastigote-containing LAMP-1^+^ phagosomes and on the inner side of their membrane. Moreover, I-A^d^/LACK complexes were present within amastigotes, a result that is in agreement with previous studies that have shown that amastigotes internalize MHC class II molecules of their host cells [17]. Lastly, I-A^d^/LACK complexes were found at the surface of small cytoplasmic LAMP-1^+^ vesicles that did not contain parasite but exhibited a diameter of 0.2–0.5 µM and for some of them, features of MVB. In macrophages, I-A^d^/LACK complexes were also present in amastigote-containing LAMP-1^+^ phagosomes and on the inner side of their membrane and in large vesicles (diameters ranging from 0.2 to 1.0 µm) in which, at least for some of them, *Leishmania* remnants could be detected, suggesting that the amastigote was being killed. In contrast to what was observed in DCs, these vesicles exhibited 2C44 staining essentially in the lumen. Finally, in eosinophils, I-A^d^/LACK complexes could only be detected in the phagosome and its membrane. This was shown both by immunogold electron microscopy following staining with 2C44 and by performing an *ex vivo* antigen presentation assay using a sensitive LACK-specific T cell hybridoma. In a recent paper, Filipe-Santos and her colleagues have examined the behavior of pathogen-specific CD4^+^ T cells at the site of *L. major* infection in BALB/c mice. Data showed that interactions between the infected phagocytes and parasite-specific CD4^+^ T cells were heterogeneous, involving both stable and dynamic contacts, and that only a fraction of infected cells induced arrest or deceleration of pathogen-specific T cells. While local variation in T cell access to infected cells could account for these observations, this phenomenon could also be the result of the absence of peptide/MHC complexes at the surface of some infected cells as we have observed in the present study.

It is tempting to speculate why some phagocytes are infected with *L. major* but do not present parasite peptides bound to MHC class II at their surface. In agreement with other studies that showed that macrophages/monocytes could process antigens and assemble peptide/MHC complexes in intracellular compartments, we found that macrophages infected with *L. major* exhibited I-A^d^/LACK complexes on the phagosome membrane. However, these complexes were not found on the plasma membrane suggesting that *L. major* has developed escape mechanisms preventing the translocation of these complexes from the phagosome to the cell surface. Several years ago, Antoine and his colleagues showed in bone marrow-derived macrophages that MHC class II molecules were retained in the phagolysosome and part of these molecules were internalized by amastigotes preventing *L. major*-infected cells to present parasite as well as exogenous antigens [16], [17]. While this phenomenon could explain the absence of I-A^d^/LACK complexes on the plasma membrane on DsRed^+^ macrophage/monocyte, it should be noticed that we readily detected I-A^d^/LACK complexes on the membrane of vesicles that, at least for some of them,exhibited features of MVB including a round to oval shape with smaller vesicles inside and a double membrane. Therefore, it is tempting to speculate that *L. major* has evolved an additional escape mechanism preventing the translocation of peptide/MHC complexes from the MVB to the cell surface.

Are I-A^d^/LACK complexes assembled in amastigote-containing phagosomes *in vivo*? Although our results unambiguously show that I-A^d^/LACK complexes are present in the phagosome of infected phagocytes, it remains possible that LACK or LACK-derived peptides are translocated from the phagosome to other cellular compartments and eventually loaded onto I-A^d^ molecules before being transported to the phagosome membrane. We do not favor this latter hypothesis for two reasons. First, phagosomes contain both MHC molecules and H2-M that is required for the loading of peptides onto MHC class II molecules [15]. Secondly, in eosinophils, I-A^d^/LACK complexes were only detected in the phagosome and not in other cellular compartments. Therefore, this result suggests that I-A^d^/LACK complexes are assembled in amastigote-containing phagosomes before being transported to the cell surface in DCs. In agreement with this hypothesis, we found that the cytoplasm of infected DCs contained 2C44^+^ vesicles and that some of them were similar to the long tubular organelles that have been described in DCs upon maturation [28]. According to this latter study, these vesicles could mediate transport to the plasma membrane.

It is of interest that I-A^d^/LACK complexes could be detected at the cell surface since LACK accounts for only 0.01% of the total amount of proteins expressed by amastigotes. This phenomenon is likely to be related to the very long half time life of the I-A^d^/LACK complexes. Our results may also suggest that I-A^d^/LACK complexes account for a significant proportion of surface peptide/MHC complexes in amastigote-containing DCs, possibly explaining why this parasite antigen is the main target of the CD4^+^ T cell response directed to *L. major* [18].

Several mAbs recognizing peptides bound to MHC class II molecules have been described. A set of mAbs, all IgM, were produced against peptides from the rat myelin basic protein bound to I-A^s^ [29]. Aw3.18 and C4H3 both reacted to HEL peptides bound to I-A^k^ [30], [31], [32], 30.2 recognized a fragment of human invariant chain (CLIP) bound to I-A^b^ [33] and Yae reacted to a peptide from the I-Eα chain bound to I-A^b^ [34], [35], [36], [37]. Although these mAbs were used in some studies, they cross-reacted with MHC molecules that were loaded with peptides other than the desired fragment of the model antigen. For example, C4H3 bound to a subset of I-A^k^ molecules loaded with certain self-peptides, and therefore the absolute background of C4H3 staining was influenced by the total amount of I-A^k^ present on the APC [38]. In this study, we have generated four mAb that were identified on the basis of their ability to bind to I-A^d^-expressing fibroblasts loaded with LACK peptide. Two out of these four mAbs bound to DCs from LACK-immunized mice, but also to DCs from OVA-immunized animals precluding their use to identify the cells that presented LACK *in vivo*. In striking contrast, 2C44 only bound to DCs from LACK-immunized mice and therefore, was suitable to identify the APCs that presented LACK *in vivo*. Furthermore, binding of 2C44 to I-A^d^/LACK was characterized by a low equilibrium dissociation constant of 1.1 nM as demonstrated by BIAcore analysis. Although the affinity as a thermodynamic parameter is not necessarily related to the specificity of a mAb, we can speculate that the exquisite specificity of 2C44 as compared to 2E60, 2F74 and 2X8 is due to recognition of a higher number of atoms of the I-A^d^/LACK complex, leading to a better stabilization of the antibody-peptide/MHC complex.

MHC class II molecules, including I-A^d^, exhibit an open groove that can accommodate antigenic peptides of different lengths [39]. Furthermore, analyzing naturally processed antigenic peptides using mass spectroscopy revealed that MHC class II-bound peptides could exhibit the same core but different amino- or carboxy-terminal extremities. Use of truncated peptides indicated the binding core of LACK_156–173_ to be residues 163–171 and suggested the following amino acid register from P1 to P9: EHPIVVSGS with E_163_, I_166_, V_168_ and S_171_ being the anchor residues [20]. In agreement with this hypothesis, LACK-specific LMR7.5 hybridomas readily secreted IL-2 when incubated with LACK_161–172_ and syngeneic APCs and LACK_161–173_ bound to I-A^d^ with high avidity. Furthermore, substitution of I_166_ by an alanine resulted in a dramatic decrease in the half life time of the I-A^d^/LACK_161–173_ complexes. Characterization of 2C44 showed that its binding to I-A^d^/LACK complexes was not affected by the removal of C_157_, but completely prevented by deletion of F_158_. Because all amastigote-containing DCs were stained with 2C44, this suggested that naturally processed LACK peptides had retained F_158_. Therefore, whatever proteases are involved in the processing of LACK by amastigote-containing DCs, trimming of the LACK peptide is limited to a minimal peptide containing F_158_ further suggesting that this peptide is protruding from the I-A^d^ peptide-binding groove by at least 5 amino acids on the amino-terminal side.

Participation of amino-terminal flanking residues in the hydrogen-bonding network stabilizing the complex has been observed for multiple peptide–class II MHC complexes from both human and mouse, and appears to be a universal feature of this interaction [40]. Furthermore, interactions involving amino-terminal flanking residues have been shown to confer resistance to editing by H2-DM in peritoneal macrophages incubated with liposome-encapsulated HEL peptides [41]. As H2-DM is present in the membrane of amastigote-containing phagosomes [42], our observation that the LACK peptide is protruding from the I-A^d^ peptide-binding groove on the amino-terminal side both on the phagosome membrane and at the cell surface is consistent with a protective role of this region to H2-DM editing of parasite antigens *in vivo*.

Monoclonal antibodies to MHC-peptide complexes have been widely used to study either antigen presentation *in vitro* or in mice immunized with recombinant antigens. To our knowledge, this study is the first one in which such a reagent was successfully used to study the presentation of a non-recombinant parasite antigen in an infectious disease model. Most importantly, the unique features of the 2C44 mAb allowed us to gain insight into the molecular and cellular mechanisms that are involved in the processing and the presentation of parasite antigen *in vivo*.

## Materials and Methods

### Ethics statement

Animal work has been conducted according to recommendations of the national laboratory animal use and care committee (CNREEA). Animal experiments were all performed in our animal facility that was approved by the French Ministry of Agriculture (agreement number: B 06-152-5). The protocols on live animals were approved by the local animal ethic committee for animal care and use (comité régional d'éthique pour l'expérimation animale (CREEA) Cote d'azur).

### Mice and parasites

BALB/c (H-2^d^), BALB.B (H-2^b^), C57BL/6 (H-2^b^), NOD (H-2^g7^), C3H (H-2^k^) mice, were purchased from Harlan (Bicester, UK). Mice were housed under Specific Pathogen Free (SPF) conditions and used between 6 and 10 weeks of age. 16.2β mice were crossed to BALB/c mice for 12 generations [21]. All animal studies were conducted according to the national laws on animal care and use. *L. major* promastigotes (World Health Organization strain WHOM/IR/-/173) were grown in M199 medium containing 20% FCS. DsRed-expressing promastigotes were prepared by inserting the gene coding for a DsRed variant [43] into the ribosomal RNA locus. Recombinant promastigotes were selected as previously described [22].

### Reagents, mAb and T cell hybridomas

LACK recombinant protein was purified as described [18]. OVA and RIBI were purchased from Sigma-Aldrich Chimie SARL (Lyon, France). CpG was purchased from Coley Pharmaceutical (USA). I-A^d^/LACK dimers were produced and purified as described [21]. The following mAbs were purchased from BD Biosciences (Le Pont de Claix, France): RA3-6B2, anti-B220; 2G9, anti-I-A^d^/I-E^d^, M1/70, anti-CD11b; HL3, anti-CD11c; 145-2C11, anti-CD3ε; AL21, anti-Ly-6C; 2.4G2, anti-FcR, e50-2440, Anti-Siglec-F; rb6-8c5, anti-Ly-6G; 3/23, anti-CD40; KT4.1, anti CD8α. Anti-CD3ε (clone 363.29.B) was purchased from Abcam. Anti-F4/80 mAb was obtained from the CalTag (Burlingame CA, USA). Anti-CCR3 mAb (clone 83102) and recombinant IL-2 were purchased from BD Biosciences. The peptides LACK_156–173_ (ICFSPSLEHPIVVSGSWD), OVA_323–339_, and the indicated LACK truncated peptides were synthesized by Mimotopes France (Paris, France). LACK-specific LMR7.5 T cell hybridoma [44] and A20 lymphoma B cells [45] have been previously described.

### Generation of anti-I-A^d^/LACK antibodies

240 µg of I-A^d^/LACK dimers in 200 µl of PBS were mixed with 200 µl of RIBI (Sigma-Aldrich Chimie SARL, Lyon, France) and 50 µl of this solution was injected in the footpad of 16.2β transgenic mice on days 1 and 8. Mice were immunized in the same footpad on days 15 and 18 with 5 µg of peptide/MHC dimers in PBS. Popliteal LN cells were harvested 15 h after the last immunization and fused to AG8X63 myeloma cells according to standard protocols. Cells were plated in 15 X flat-bottomed 96 well-plates in RPMI 1640 supplemented with non essential aminoacids and 10% FCS. Supernatants were screened by flow cytometry. Briefly, I-A^d^-transfected fibroblasts were pulsed for 2 h at 37°C with 20 µM of either LACK_156–173_ or OVA_323–339_. 2×10^5^ cells were washed twice with PBS containing 1% BSA, resuspended in 100 µl of supernatants and incubated for 1 h at 4°C. Cells were washed in PBS containing 1% BSA and fibroblast-bound antibodies were revealed using FITC-conjugated goat anti-mouse Ig.

### Biosensor experiments

The BIACORE 3000 system, sensor chip CM5, surfactant P20, amine coupling kit containing N-hydroxysuccinimide (NHS) and 1-ethyl-3-(3-dimethylaminopropyl)-carbodiimide (EDC) were from BIACORE (Uppsala, Sweden). Streptavidin was obtained from Sigma. All biosensor assays were performed with Hepes-Buffered Saline (HBS) as running buffer (10 mM Hepes, 150 mM NaCl, 3.4 mM EDTA, 0.02% surfactant P20, pH 7.4). Streptavidin was immobilized on CM5 sensor chips by amine coupling according the manufacturer's protocol. The amount of immobilized mAb was approximately 5 ng/mm^2^. The four biotinylated mAb were immobilized on the streptavidin chip, each on one channel, at a concentration of 10 µg/ml and at a flow rate of 10 µl/min for 1 minute. The channels were washed with 10 µl of regenerating solution (KSCN 0.51 M, citrate 0.2 M, pH = 5). The amounts of bound mAb were 2.0, 2.4, 2.1 and 1.3 ng/mm^2^ for 2F74, 2C44, 2X80 and 2E60, respectively. I-A^d^/LACK and I-A^d^/Ig dimers were used at concentrations of 50 to 400 nM and kinetic parameters were measured at 25°C. In the association phase, dimers were fluxed over the antibody chip at a flow rate of 20 µl/min for 7 minutes. In the dissociation phase, buffer alone was fluxed over the chip for 10 minutes. After each cycle, 10 µl of regenerating buffer was used to return to the start value. The kinetic parameters were calculated using the BIAeval 3.1 software. Global analysis was performed using the bivalent analyte model after subtracting the sensorgrams of the I-A^d^/Ig control dimer from that of the I-A^d^/LACK dimer. The bivalent analyte model was used since the model fitted best to the experimental values.

### Immunization and infection

Immunization was performed by injecting 6 week-old BALB/c mice in both footpads with a truncated version of LACK (40–50 µg per footpad) together with 25 µg of CpG. Cells from the draining lymph nodes were prepared 40 hours later, labeled with fluorescent antibodies and analyzed by flow cytometry. Infection was performed by injecting 6 wk-old BALB/c mice with 10^6^ purified metacyclic promastigotes in 25 µl per footpad. Mice were sacrificed 4 wk later.

### Cell purification

To prepare DC, LN were digested with a cocktail of DNase I fraction IX (Roche, diagnostics) (100 µg/ml) and 1.6 mg/ml of collagenase (400 Mandl U/ml) at 37°C for 45 min. DCs were positively selected by MACS using N418 (anti-CD11c) magnetic beads (Miltenyi Biotec, Germany) in the presence of 10% mouse serum and 5 mM EDTA according to the manufacturer instructions. Positively selected cells were >95% pure as determined by flow cytometry following staining with anti-CD11c mAb. To prepare phagocytes, LN cells were depleted of T and B cells. To this aim, cells were incubated with anti-CD3 and anti-CD19 mAb, and CD3^+^ and CD19^+^ cells were depleted by negative selection using anti-rat IgG magnetic Dynabeads (Invitrogen, France) resulting in a cell population containing 12% and 13% of B and T lymphocytes respectively (Supplementary Figure 5). Alternatively, cells were incubated with biotinylated anti-CD3 and anti-CD19 mAb, and CD3^+^ and CD19^+^ cells were depleted using streptavidin-coupled magnetic beads (Myltenyi Biotec, Germany), resulting in a cell population containing 8% and 6% of B and T lymphocytes respectively (Supplementary Figure 5).

### T cell assay

The indicated numbers of cells were incubated with 10^5^ LMR7.5 hybridomas with or without 1 µM of LACK_156–173_ in DMEM supplemented with 2 mM L-glutamine, 10% heat-inactivated FCS, 5×10^−5^ M 2-mercaptoethanol, 100 µg/ml penicillin and 100 U/ml streptomycin in the wells of U-bottomed 96 well-plates. Supernatants were harvested 24 h later, and IL-2 contents were measured by ELISA.

### Flow cytometry

Cells were analyzed by flow cytometry using a FACSCanto or a LSR II with the FACS Diva software (Becton Dickinson, San Jose, CA). Cell sorting was carried out using a FACS Aria (Becton Dickinson, San Jose, CA).

### Electron microscopy

Flow-sorted cells were primary fixed with 1.6% glutaraldehyde in 0.1 M phosphate buffer pH 7.5, then at 4°C with 1% osmium tetroxide in 100 mM cacodylate buffer pH 7.5. Pellets were washed five times in water, dehydrated in increasing acetone series and embedded in epoxy resin. 70 µM thin sections were prepared and contrasted for observation with a Philips CM12 or Jeol 1400 electron microscope.

### Immunogold electron microscopy

Immunogold labeling was performed on ultrathin cryo-sections of FACS-purified DsRed^+^ cells. Cells were fixed for 1 h at 4°C in 0.1 M phosphate buffer pH 7.5 containing 4% paraformaldehyde and 0.2% glutaraldehyde. Cells were then washed with phosphate buffer (PBS) containing 50 mM NH_4_Cl. Small blocks were infiltrated with 2.3 M sucrose, frozen in liquid nitrogen and then cut on a dry diamond knife at −120°C. Ultrathin 70 nm sections were then collected on formvar-coated nickel grids and processed for immunochemistry. Grids were deposited face down on the top of small drops of the following solutions: PBS containing 50 mM NH_4_Cl for 10 min, PBS containing 1% BSA for 5 min, PBS containing the relevant mAbs in 1% BSA for 1 h, PBS containing 1% BSA for 10 min, PBS containing 0.1% BSA for 5 min, PBS containing 1% BSA and either 10 or 15 nm colloidal gold conjugated anti-mouse IgG antibodies for 30 min, PBS containing 0.1% BSA for 5 min, PBS for 5 min twice, PBS containing 1% glutaraldehyde for 5 min and distilled water for 5 min. Grids were then embedded in methylcellulose as described elsewhere [46]. Preparations were observed with a Philips CM12 or a Jeol 1400 electron microscope operating at 80 kV equipped with CCD camera (Olympus SIS, Germany). Micrographs were systematically taken on labeling area to visualize the number of beads on the structure. No signal was observed when the first antibody was omitted.

## Supporting Information

Figure S1 Flow cytometry analysis of LN cells from immunized mice stained with different mAbs reacting to I-A^d^/LACK complexes. BALB/c mice were immunized or not with either LACK or OVA in CpG. LN cells were purified 2 days later, enriched for CD11c^+^ cells using magnetic beads and stained with either 2C44, 2F74, 2E60 or 2X8. Data show representative FACS profiles after gating on CD11c^+^ cells.
(0.06 MB TIF)

Figure S2 Lymphocyte-depleted cells from 4 wk-infected BALB/c mice were analyzed by multicolor flow cytometry. Data show representative profiles after gating successively on live cells (upper left panel), CD11b^high^ CD11c^−^ cells (G1, upper right panel), Ly-6G^int^ Ly-6C^int^ (G2, lower left panel) and Siglec-F^high^ CCR3^+^ cells (G3, lower right panel). The frequency of DsRed^+^ cells in the gated population is indicated.
(0.31 MB TIF)

Figure S3 Flow cytometry analysis of DCs, macrophages/monocytes and neutrophils. Lymphocyte-depleted cells from 4 wk-infected BALB/c mice were analyzed by multicolor flow cytometry after gating out eosinophils (R2 gate defined in Figure 3). (A) DCs. Data show representative profiles before (left panel) and after gating on G1 (right panel). (B) Macrophages/monocytes. Data show representative profiles before (left panel) and after gating successively on G2 (middle panel) and G3 (right panel). (C) Neutrophils. Data show representative profiles before (left panel) and after gating successively on G4 (middle panel) and G5 (right panel). The frequency of DsRed^+^ cells in the gated population is indicated.
(0.46 MB TIF)

Figure S4 High-resolution electron microscopy analysis of amastigote-containing phagosomes. Data show enlargments of DC phagosomes. The left photograph shows 2 phagosomes with the parasite nucleus, membrane and cortical microtubules. The right photograph shows a high magnification view of the phagosomal and amastigote membranes. P: phagosome; C: cytoplasm.
(2.70 MB TIF)

Figure S5 Flow cytometry analysis of LN cells before and after depletion of lymphocytes. Lymphocyte-depleted cells from 4 wk-infected BALB/c mice were analyzed by flow cytometry before (A) or after depletion of CD3^+^ and CD19^+^ lymphocytes using streptavidin-coupled beads (B) or anti-rat Ig Dynabeads (C). Data show representative FACS profiles after staining with anti-B220 (left panels) or anti-CD3 (right panels) mAbs. Of note, two different clones of anti-CD3 mAb were used for depletion (145-2C11) and staining (C363.29.B). The frequency of cells that are stained with the indicated mAb is shown.
(0.15 MB TIF)

Table S1 Kinetics parameters of the binding of different mAbs to I-A^d^/LACK dimer. The indicated mAbs were biotinylated and immobilized on a streptavidin chip that was fluxed with either I-A^d^/LACK dimers or I-A^d^/Ig control dimers. The kinetics parameters were calculated using the BIAeval 3.1 software. Global analysis was performed using the bivalent analyte model after subtracting the sensorgrams of the I-A^d^/Ig control dimer from that of the I-A^d^/LACK dimer.
(0.03 MB DOC)
