# Supplementary figures and images for: Direct Visualization of Peptide/MHC Complexes at the Surface and in the Intracellular Compartments of Cells Infected In Vivo by Leishmania major
Source: PLoS Pathog. 2010 Oct 14;6(10):e1001154. doi: 10.1371/journal.ppat.1001154 (PMC2954901; doi:10.1371/journal.ppat.1001154)

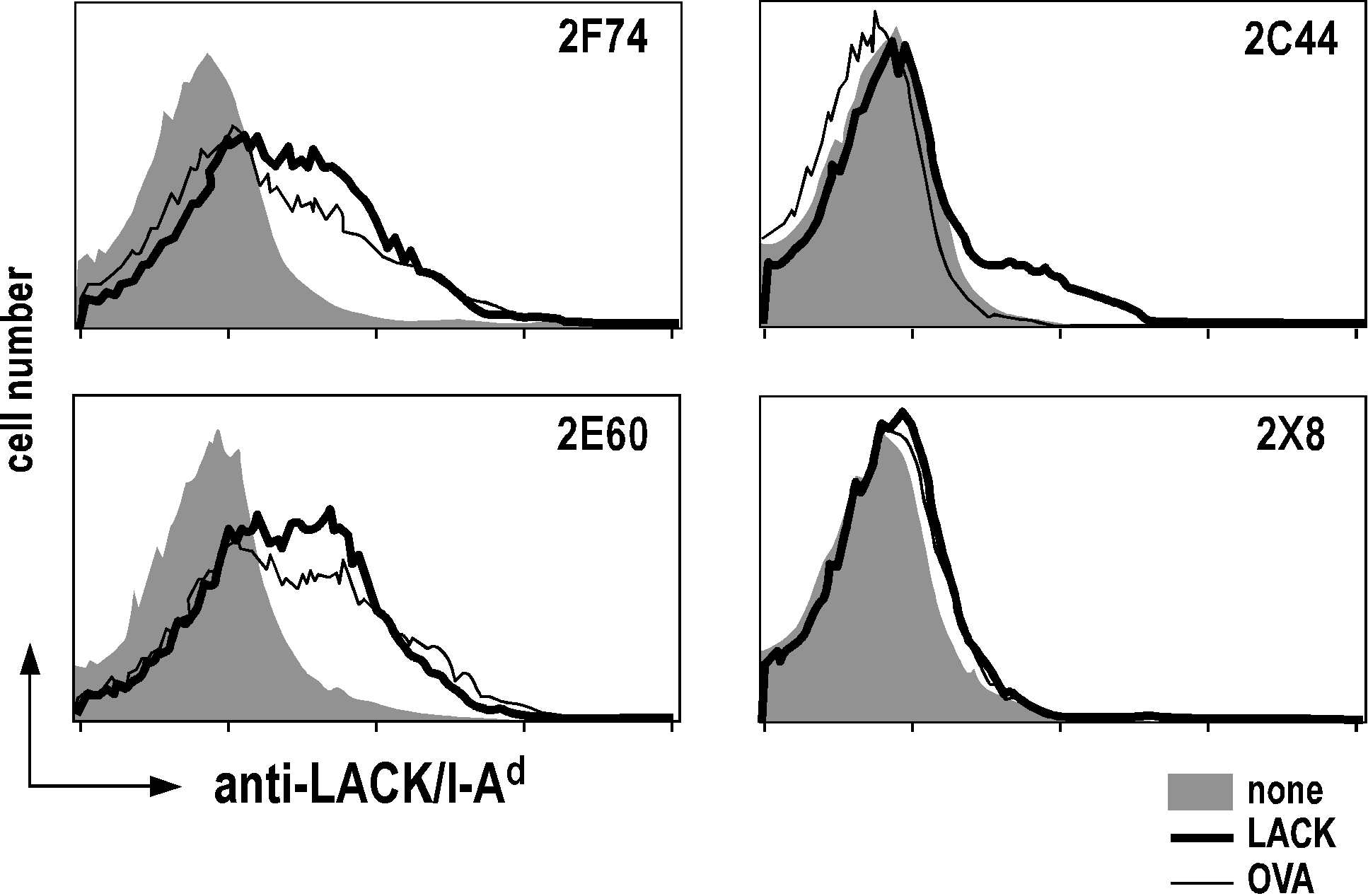

Supplement: Figure S1 — Flow cytometry analysis of LN cells from immunized mice stained with different mAbs reacting to I-Ad/LACK complexes. BALB/c mice were immunized or not with either LACK or OVA in CpG. LN cells were purified 2 days later, enriched for CD11c+ cells using magnetic beads and stained with either 2C44, 2F74, 2E60 or 2X8. Data show representative FACS profiles after gating on CD11c+ cells. (0.06 MB TIF) [file ppat.1001154.s001.tif]

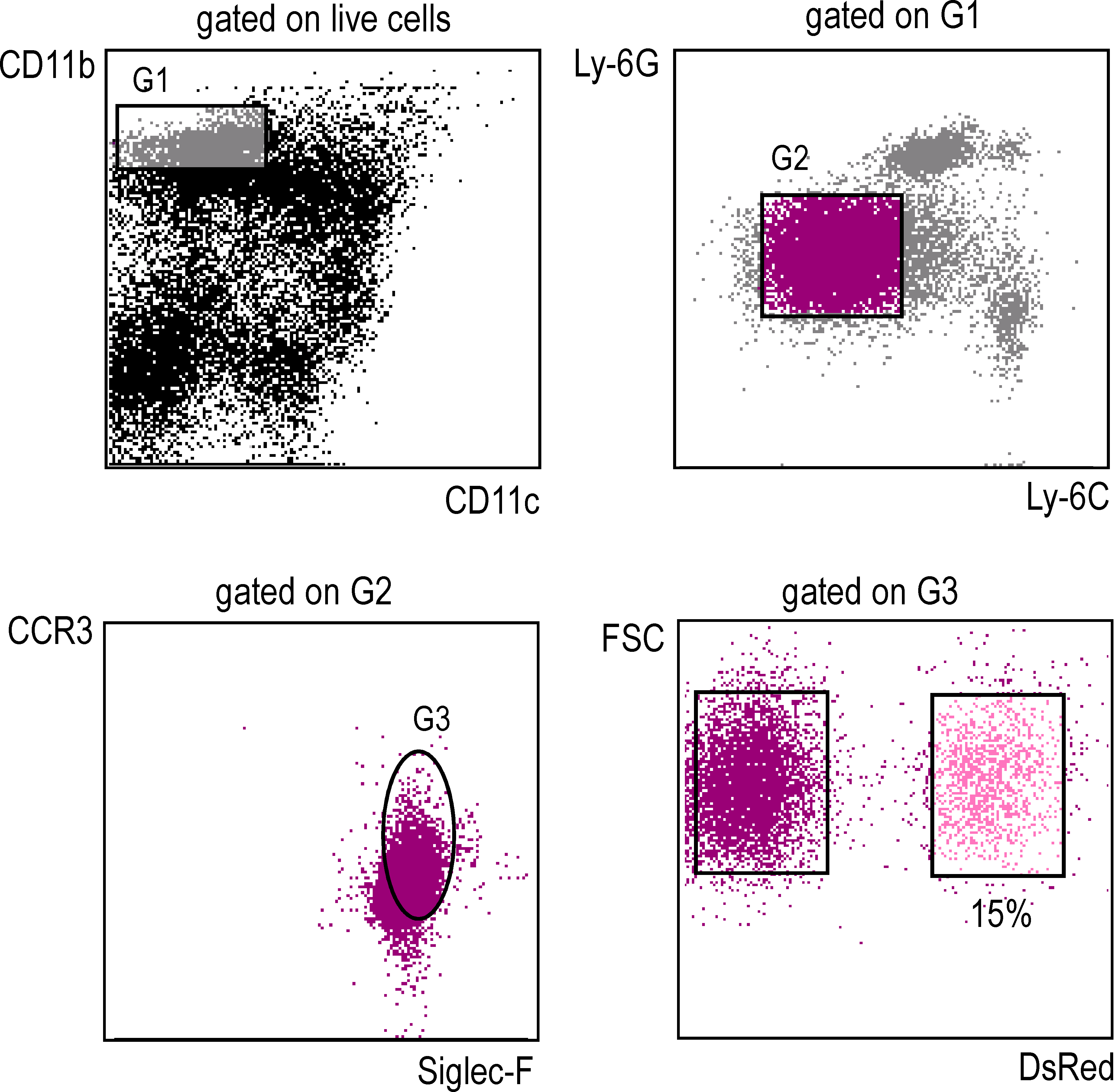

Supplement: Figure S2 — Lymphocyte-depleted cells from 4 wk-infected BALB/c mice were analyzed by multicolor flow cytometry. Data show representative profiles after gating successively on live cells (upper left panel), CD11bhigh CD11c− cells (G1, upper right panel), Ly-6Gint Ly-6Cint (G2, lower left panel) and Siglec-Fhigh CCR3+ cells (G3, lower right panel). The frequency of DsRed+ cells in the gated population is indicated. (0.31 MB TIF) [file ppat.1001154.s002.tif]

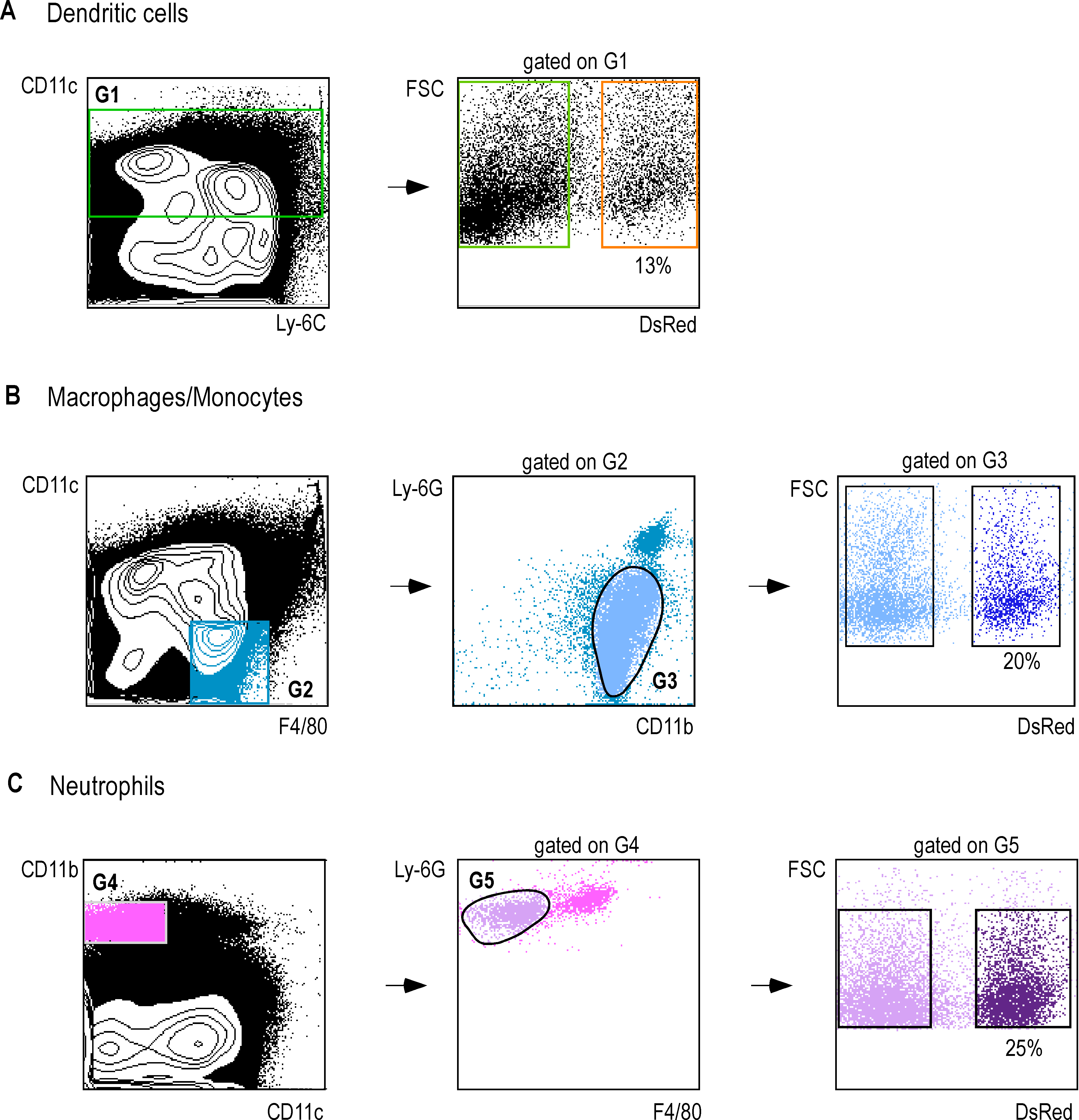

Supplement: Figure S3 — Flow cytometry analysis of DCs, macrophages/monocytes and neutrophils. Lymphocyte-depleted cells from 4 wk-infected BALB/c mice were analyzed by multicolor flow cytometry after gating out eosinophils (R2 gate defined in Figure 3). (A) DCs. Data show representative profiles before (left panel) and after gating on G1 (right panel). (B) Macrophages/monocytes. Data show representative profiles before (left panel) and after gating successively on G2 (middle panel) and G3 (right panel). (C) Neutrophils. Data show representative profiles before (left panel) and after gating successively on G4 (middle panel) and G5 (right panel). The frequency of DsRed+ cells in the gated population is indicated. (0.46 MB TIF) [file ppat.1001154.s003.tif]

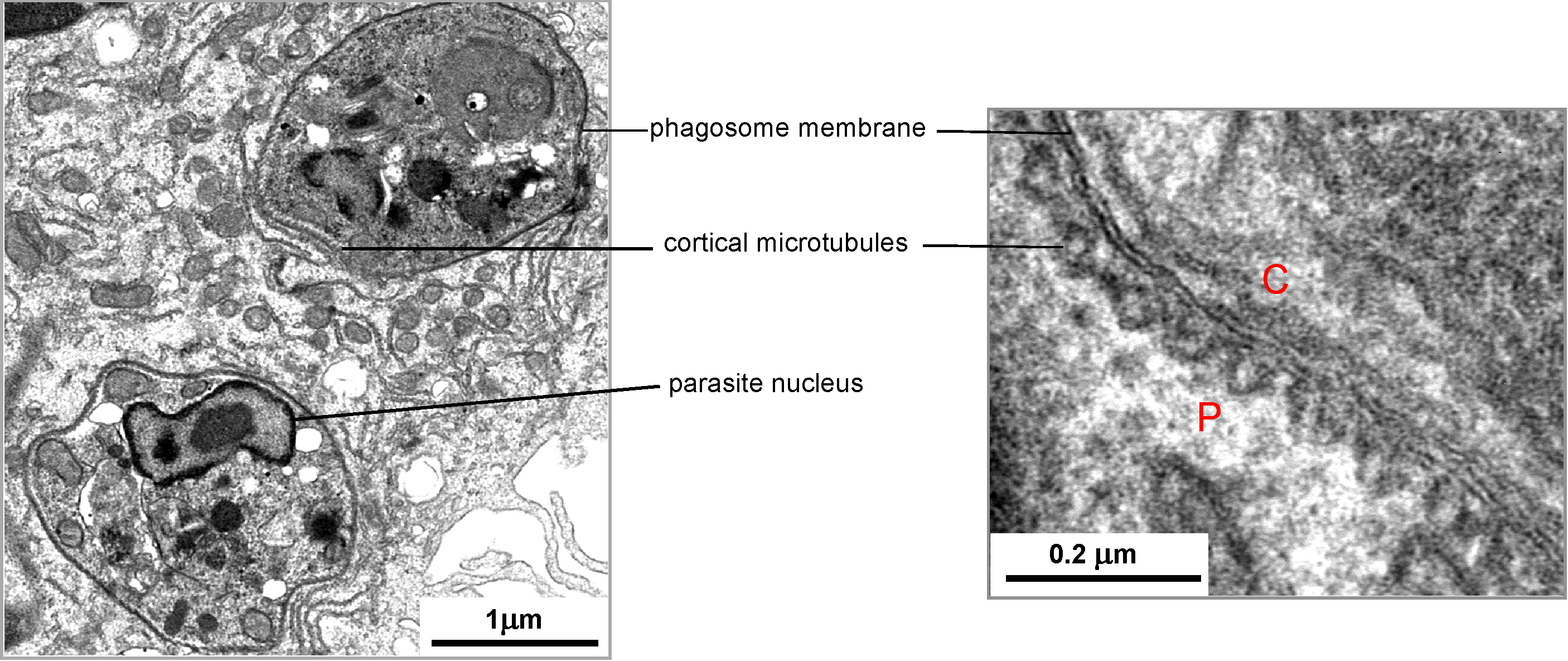

Supplement: Figure S4 — High-resolution electron microscopy analysis of amastigote-containing phagosomes. Data show enlargments of DC phagosomes. The left photograph shows 2 phagosomes with the parasite nucleus, membrane and cortical microtubules. The right photograph shows a high magnification view of the phagosomal and amastigote membranes. P: phagosome; C: cytoplasm. (2.70 MB TIF) [file ppat.1001154.s004.tif]

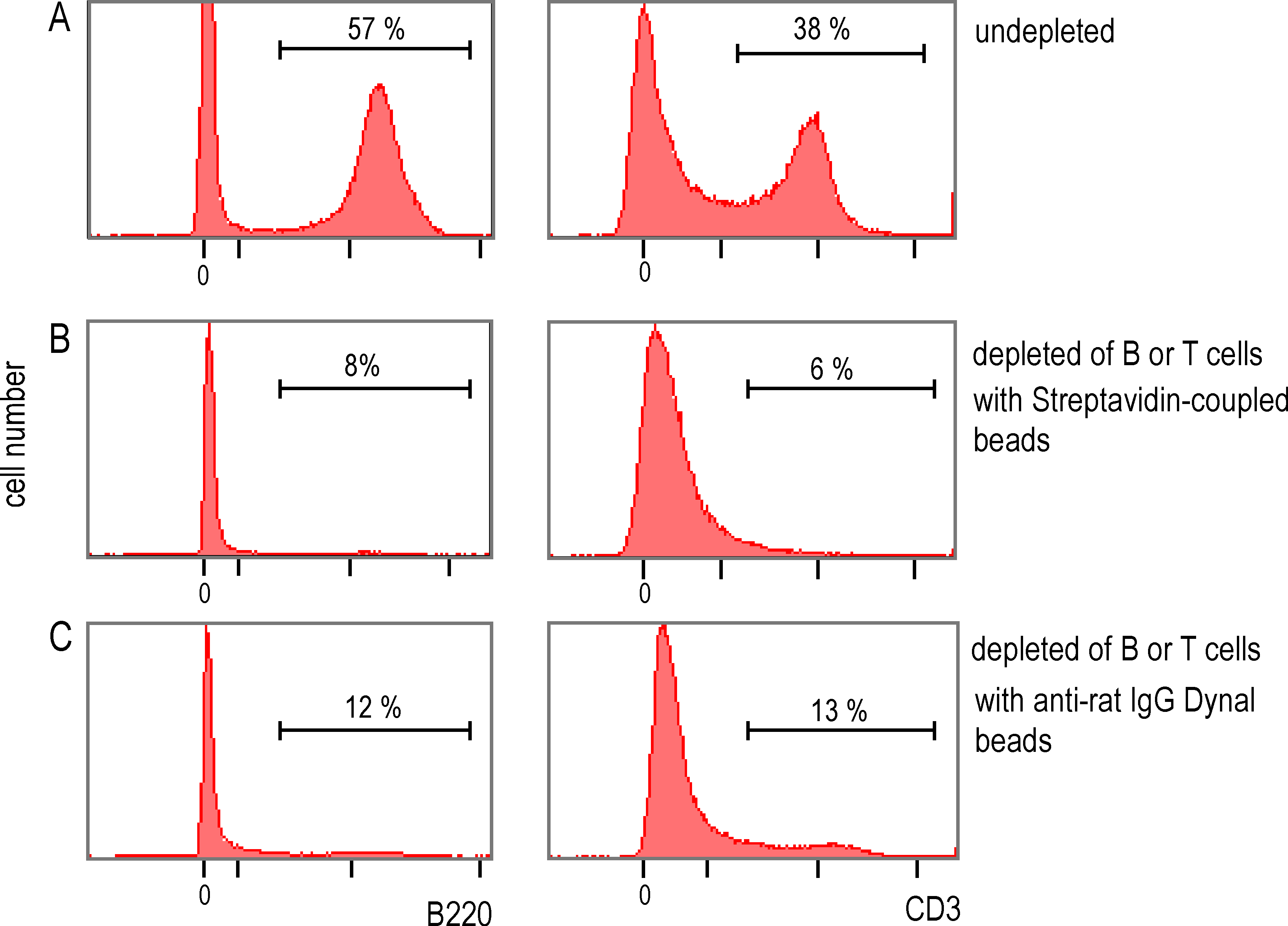

Supplement: Figure S5 — Flow cytometry analysis of LN cells before and after depletion of lymphocytes. Lymphocyte-depleted cells from 4 wk-infected BALB/c mice were analyzed by flow cytometry before (A) or after depletion of CD3+ and CD19+ lymphocytes using streptavidin-coupled beads (B) or anti-rat Ig Dynabeads (C). Data show representative FACS profiles after staining with anti-B220 (left panels) or anti-CD3 (right panels) mAbs. Of note, two different clones of anti-CD3 mAb were used for depletion (145-2C11) and staining (C363.29.B). The frequency of cells that are stained with the indicated mAb is shown. (0.15 MB TIF) [file ppat.1001154.s005.tif]
