# Supplementary material for: Direct Visualization of Peptide/MHC Complexes at the Surface and in the Intracellular Compartments of Cells Infected In Vivo by Leishmania major
Source: PLoS Pathog. 2010 Oct 14;6(10):e1001154. doi: 10.1371/journal.ppat.1001154 (PMC2954901; doi:10.1371/journal.ppat.1001154)
Supplement: Table S1 — Kinetics parameters of the binding of different mAbs to I-Ad/LACK dimer. The indicated mAbs were biotinylated and immobilized on a streptavidin chip that was fluxed with either I-Ad/LACK dimers or I-Ad/Ig control dimers. The kinetics parameters were calculated using the BIAeval 3.1 software. Global analysis was performed using the bivalent analyte model after subtracting the sensorgrams of the I-Ad/Ig control dimer from that of the I-Ad/LACK dimer. (0.03 MB DOC) [file ppat.1001154.s006.doc]

| mAb | k_1_ (M^-1^ s^-1^) x 10^4^ | k _-1_ (s^-1^) x 10^-3^ | K_D_ = k _-1_ / k_1_  (M) |
| --- | --- | --- | --- |
| 2C44 | 6.64 | 0.076 | 1.1 x 10^-9^ |
| 2F74 | 4.38 | 5.19 | 1.2 x 10^-7^ |
| 2E60 | 4.94 | 0.98 | 2.0 x 10^-8^ |
| 2X8 | 3.94 | 0.17 | 4.3 x 10^-9^ |
